# Allele-selective targeting of uromodulin by antisense oligonucleotides reduces aggregates and kidney damage in a mouse model of autosomal dominant tubulointerstitial kidney disease

**DOI:** 10.1093/nar/gkag1007

**Published:** 2026-11-10

**Authors:** Sebastian Sjöström, Marta Mariniello, Guglielmo Schiano, Jennifer Lake, Sara Botta, Alyssa Hill, Jonathan Hall, Olivier Devuyst

**Affiliations:** Institute of Pharmaceutical Sciences, Department of Chemistry and Applied Biosciences, ETH Zurich, 8093 Zurich, Switzerland; Mechanisms of Inherited Kidney Disorders, Institute of Physiology, University of Zurich, 8057 Zurich, Switzerland; Mechanisms of Inherited Kidney Disorders, Institute of Physiology, University of Zurich, 8057 Zurich, Switzerland; Mechanisms of Inherited Kidney Disorders, Institute of Physiology, University of Zurich, 8057 Zurich, Switzerland; Mechanisms of Inherited Kidney Disorders, Institute of Physiology, University of Zurich, 8057 Zurich, Switzerland; Institute of Pharmaceutical Sciences, Department of Chemistry and Applied Biosciences, ETH Zurich, 8093 Zurich, Switzerland; Institute of Pharmaceutical Sciences, Department of Chemistry and Applied Biosciences, ETH Zurich, 8093 Zurich, Switzerland; Mechanisms of Inherited Kidney Disorders, Institute of Physiology, University of Zurich, 8057 Zurich, Switzerland; Institute for Rare Diseases, Saint-Luc Academic Hospital, UCLouvain, 1200 Brussels, Belgium

## Abstract

Autosomal dominant tubulointerstitial kidney disease (ADTKD) arises from gain-of-toxic-function mutations in the *UMOD* gene coding for uromodulin, the most abundant protein excreted in normal urine. Mutant uromodulin accumulates in the cells lining the thick ascending limb (TAL) of the loop of Henle, leading to intracellular aggregates, cellular stress, and kidney damage. Here, we demonstrate that an antisense gapmer oligonucleotide (ASO) designed against the representative c.553C > A (p.R185S) *UMOD* mutation produced allele-selective silencing of the mutant messenger RNA and protein in kidney cells. Short-term administration of a murine ASO analogue in the established *Umod*  ^R186S/+^ mouse model of ADTKD suppressed uromodulin and lowered tubular damage, whereas a non-targeting scrambled control was inactive. After a 4-week treatment, ASO accumulation in the *Umod*  ^R186S/+^ kidneys reached ∼320 000 copies per TAL cell, quantified using a sensitive splint-ligation assay. The ASO treatment suppressed premature mutant uromodulin and its toxic aggregates in *Umod*  ^R186S/+^ kidneys, whereas it had no effects on wild-type uromodulin in *Umod*  ^+/+^ kidneys. Notably, the ASO improved tubular function and decreased kidney tubulointerstitial inflammation in *Umod*  ^R186S/+^ mice. This work offers a roadmap for antisense therapy to treat ADTKD-*UMOD* and substantiates the therapeutic potential of ASOs in disorders of the kidney tubule.

## Graphical Abstract

**Graphical Abstract:**
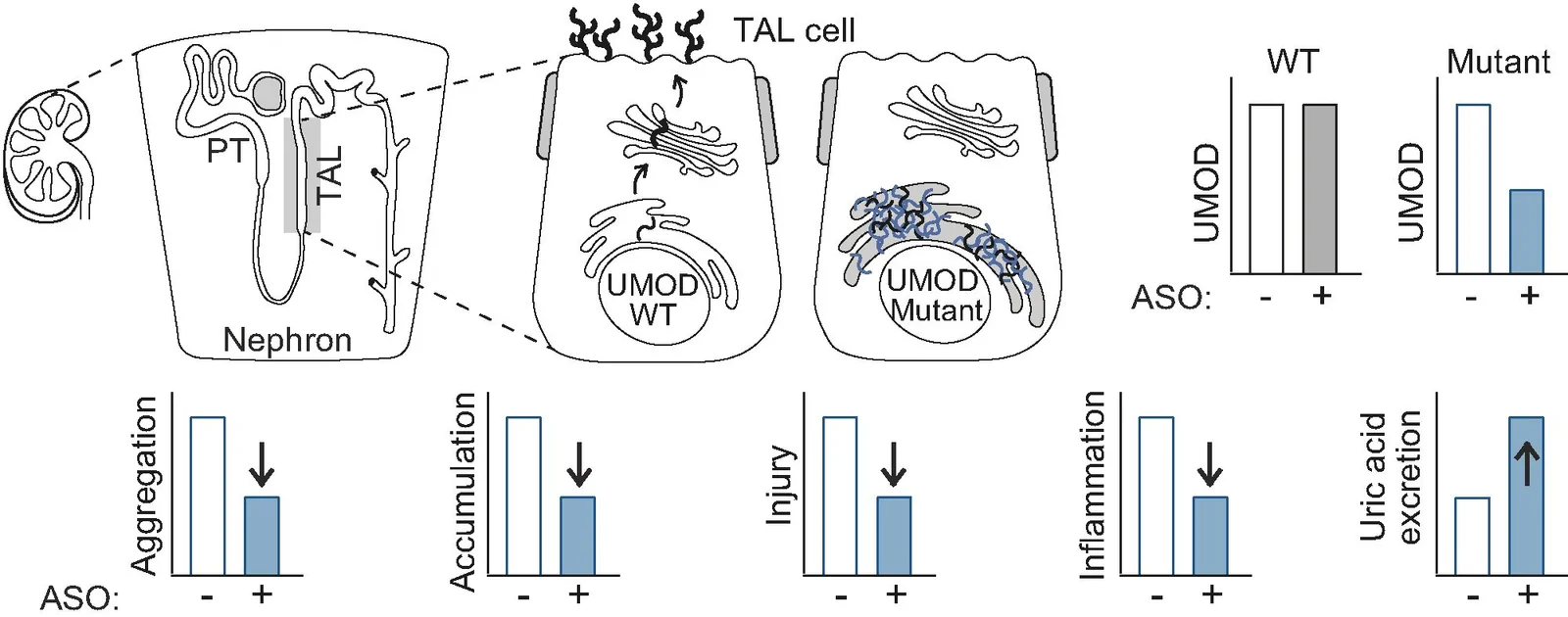

## Introduction

Autosomal dominant tubulointerstitial kidney disease (ADTKD) includes a set of monogenic kidney disorders that cause chronic kidney disease and invariably progress to kidney failure. There is currently no specific treatment to alter the progression of ADTKD, one of the most frequent types of monogenic kidney disease [1]. The most common gene causing ADTKD is *UMOD*, which is exclusively expressed in the kidney and encodes uromodulin, a highly abundant protein excreted in the urine [2]. Uromodulin is a glycoprotein, mainly produced by cells lining the thick ascending limb (TAL) of the loop of Henle [3]. After its maturation in the endoplasmic reticulum (ER) and Golgi, uromodulin is trafficked to the apical plasma membrane, where it is released into the tubular lumen to form high‐molecular‐weight polymers [4]. Uromodulin exerts important roles in tubular cells and lumen, including regulation of sodium transport [5, 6], inhibition of kidney stone formation [7], and protection against urinary tract infections [8, 9]. However, the absence of uromodulin expression does not lead to overt kidney damage: *Umod* knock-out mice only show mild manifestations of salt loss, with normal growth and no kidney damage—even in old age [10].

Most of the *UMOD* mutations causing ADTKD are missense variants that impair the maturation and trafficking of uromodulin and lead to formation of toxic aggregates in the ER of TAL cells. In turn, these aggregates activate ER stress and unfolded protein and immune responses, leading to kidney damage [1]. Reflecting this abnormal processing, a dramatic reduction in uromodulin levels in the urine is consistently observed in individuals with ADTKD‐*UMOD* [11, 12]. Recent studies in kidney cells and knock-in mice carrying the representative missense p.R185S *UMOD* mutation recapitulated the disease course and demonstrated the role of toxic aggregates in causing tubulointerstitial damage and fibrosis—a paradigm of toxic proteinopathy [13]. Notably, these studies also showed that wild-type (WT) uromodulin partly rescues the mutant uromodulin trafficking and protects against the formation of toxic uromodulin aggregates [13].

Antisense oligonucleotides (ASOs) and small interfering RNAs are a rapidly growing class of drugs that are well adapted to the treatment of rare genetic diseases. The majority of the 22 FDA-approved drugs treat disorders by suppressing a target messenger RNA (mRNA) in the liver, while the remainder modulate mRNA splicing and restore gene expression in the eye, muscle, or nervous system [14]. Upon systemic administration, ASOs distribute broadly and accumulate in the kidneys [15], where they show good tolerability [16]. Yet, thus far, there are no approved oligonucleotides that target the kidney, although ASOs have been investigated for autosomal dominant polycystic kidney disease [17, 18] and Alport syndrome [19]. Notably, oligonucleotide therapeutics targeting proteinopathies are advancing, with several candidates in development [20], and the recent approval of tofersen for amyotrophic lateral sclerosis [21].

Most approved ASOs are chimeric “gapmer” oligonucleotides that carry riboses and internucleotide linkages modified with 2′-*O*-methoxyethyl (MOE) and phosphorothioate (PS) groups, respectively [22]. The ASOs localize predominantly in cell nuclei, where they hybridize to their target mRNA and induce its degradation by RNase H. The degree of selectivity that can be attained by a gapmer ASO for a mutated mRNA sequence over its WT variant depends on many factors. Since WT uromodulin mitigates the formation of toxic aggregates and the progression of ADTKD [13], selective inhibition of the mutant *UMOD* allele would therefore be preferred for an oligonucleotide-based therapy, akin to other types of dominant proteinopathies [23].

Here, we evaluated the feasibility of allele-selective ASO therapy in ADTKD-*UMOD*, using established kidney cell and knock-in (KI) mouse models of the disease. We designed and characterized allele-selective PS-MOE gapmer ASOs against mutated human and murine uromodulin for kidney-targeted delivery. We quantified ASO trafficking to specific kidney tubular cells in *Umod*  ^R186S/+^ and *Umod*  ^+/+^ mice, using a novel splint-ligation quantitative polymerase chain reaction (qPCR) assay. We showed that the lead ASO achieves allele-selective suppression of mutant uromodulin, while sparing the WT copy. Its on-target activity was confirmed using a non-targeting scrambled control, which was inactive against mutant uromodulin and downstream markers of cellular and tubular stress. In a 4-week study, we demonstrated that ASO treatment reduces mutant uromodulin accumulation and mitigates downstream tissue injury, improving kidney health in *Umod*  ^R186S/+^ mice. The lead ASO showed no effects on WT uromodulin in *Umod*  ^+/+^ mice, supporting its allele-selective targeting. These findings pave the way for translational studies using ASOs to target toxic proteinopathies in the kidney and offer a promising strategy for treating ADTKD-*UMOD*.

## Materials and methods

### Uromodulin sequences

Mature mRNA sequence of human *UMOD-*R185S*-*EGFP (Supplementary Figs S1 and S2), expressed by the mouse inner medullary collecting duct (mIMCD-3) kidney tubule cell system [13], was used for the generation of the ASO library (Supplementary Table S1). The expression construct contains EGFP and the pathological mutation c.553A (p.R185S). A WT variant containing the canonical c.553C was used for counter-screening (Fig. 1). For the generation of mA6, the sequence was compared with public reference gene *Mus musculus Umod*, transcript variant 2, mRNA (Ref. seq. NM_001278605.1) from the National Center for Biotechnology Information, and analysis was carried out in CLC Genomics Workbench 12 (Qiagen).

**Figure 1.**
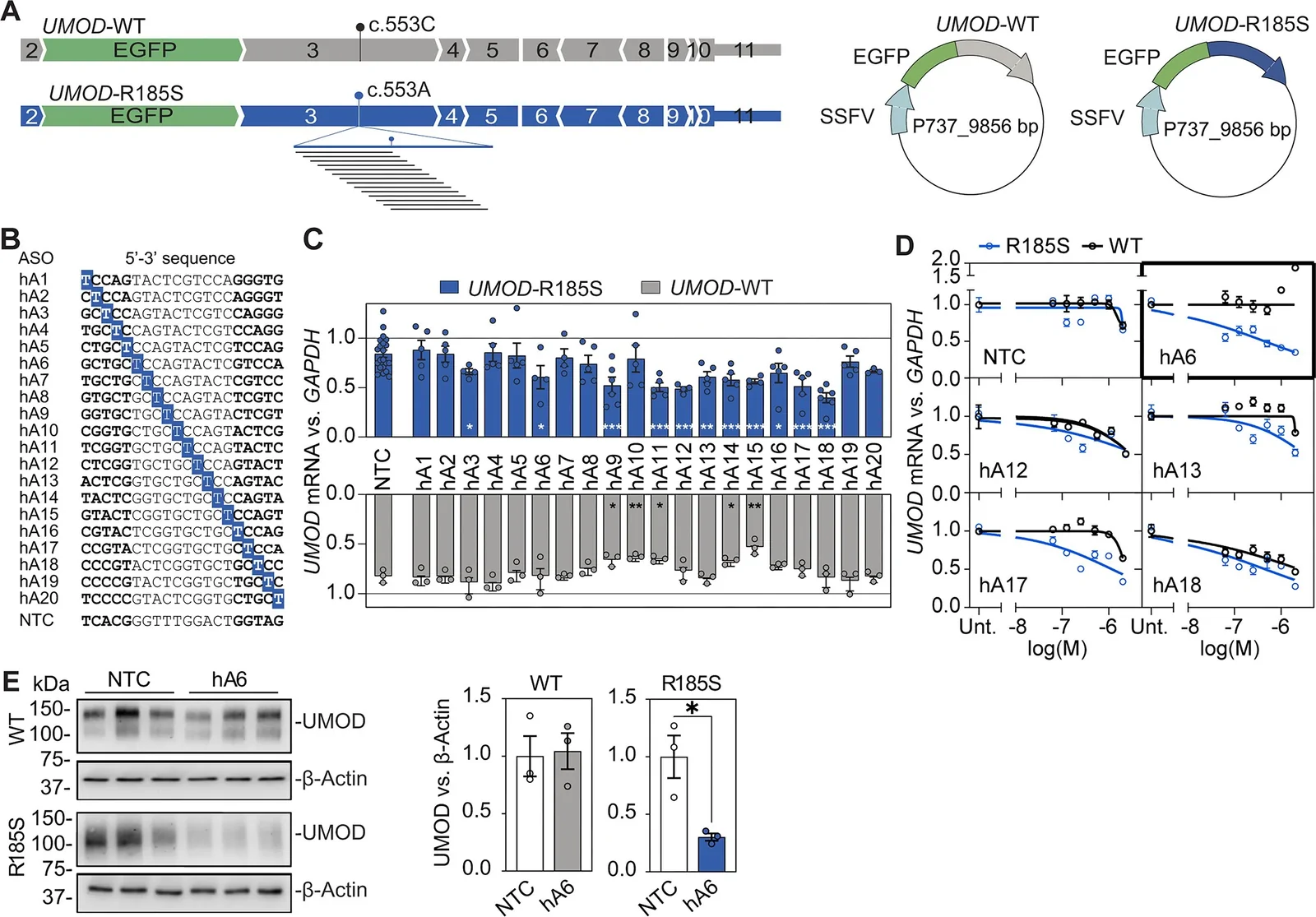
Identification of allele-selective ASOs against human *UMOD* mRNA and protein. (**A**) Engineered human *UMOD*-EGFP fusion constructs carrying the pathogenic mutation (*UMOD*-R185S) or WT (*UMOD*-WT) inserted into a vector for stable expression by mIMCD-3 cells. (**B**) ASO sequence library for targeting of the pathogenic c.553C > A (p.R185S) site. The T nucleobase opposite the mutated A is indicated with blue shading, and bold letters represent 2′MOE-modified nucleotides. Non-targeting control (NTC) is a sequence-scrambled, non-targeting negative control with identical chemical modifications to library members. (**C**) Relative *UMOD* mRNA from mIMCD-3 cells expressing mutant (blue) or WT (gray) *UMOD* following 48 h transfection with 100 nM ASO, quantified by reverse transcription quantitative polymerase chain reaction (RT-qPCR). (**D**) ASO concentration-escalation (63–2000 nM), silencing of *UMOD*-R185S (blue circles) or *UMOD*-WT (black circles), of five transfected ASOs with acceptable on/off target activity, for 48 h – ASO hA6 highlighted. Quantified by RT-qPCR; values are normalized to dose-equivalent lipofectamine vehicle treatment. (**E**) Western blot analysis for uromodulin (UMOD) and β-actin, and quantification by blot densitometry of mIMCD-3 cells following transfection of 1.6 µM hA6 or NTC for 72 h. Data expressed as means (*N* ≥ 3) ±SEM; *P < .05, **P < .01, and ***P < .001 indicate *t*-test comparisons with an NTC ASO.

### Cell models

The mIMCD‐3 kidney tubular cells (ATCC CRL‐2123) were transduced with lentiviral particles encoding EGFP‐tagged human uromodulin isoforms WT and R185S mutant. Site‐directed mutagenesis was performed using the QuikChange Lightning Kit (Stratagene, La Jolla, CA) as described previously [13]. The EGFP sequence was inserted downstream of the leader peptide (first 25 amino acids). Following lentiviral transduction, cells were sorted based on EGFP expression using a BD Aria III cell sorter (Cytometry Facility, University of Zurich, Switzerland). The cells were cultured in Iscove’s modified Dulbecco’s medium (IMDM, Thermo Fisher Scientific, Waltham, MA, USA) supplemented with 10% fetal bovine serum (Thermo Fisher Scientific) and 1% penicillin/streptomycin (Thermo Fisher Scientific). The cells were authenticated and tested for mycoplasma contamination using the EZ-PCR Mycoplasma Detection Kit (Sartorius).

### Isolation and treatment of mouse thick ascending limbs

TALs were isolated from 28-day-old male *Umod*  ^R186S/+^ mice as previously described [24]. Kidneys were rapidly excised and placed in ice-cold HBSS dissection solution (Lonza, Verviers, Belgium) supplemented with 15 mM HEPES (Lonza), 10 mM D-glucose (VWR International, Lucerne, Switzerland), 5 mM glycine (VWR International), and 1 mM L-alanine (Applichem GmbH, Darmstadt, Germany), adjusted to pH 7.4. Each kidney was bisected along the midsagittal plane and further sectioned transversely. After removal of the cortex and inner medulla, the outer medulla was dissected into ∼1 mm³ pieces. Tissue fragments were incubated for 30 min in dissection solution containing 245 U/ml type II collagenase (Worthington Biochemical Corp., Lakewood, USA) and 96 μg/ml soybean trypsin inhibitor (Sigma, St. Louis, USA). Following enzymatic digestion, the suspension was passed through a 250 μm mesh filter, and tubular fragments were collected on an 80 μm filter to enrich for tubules longer than 100 μm. The retained tubules were resuspended in pre-warmed (37°C) albumin solution (dissection solution supplemented with 1% [w/v] bovine serum albumin and 96 μg/ml soybean trypsin inhibitor). TAL segments were identified based on morphological criteria under a light microscope (Leica DM IL, Bensheim, Germany) and manually isolated using a glass pipette mounted on a micromanipulator (Narishige International, London, UK). Approximately 50 TAL segments were pooled per sample and transferred into 1.5 ml microcentrifuge tubes. TALs were incubated with either 100 or 200 nM mA6 or NTC for 48 h at 37°C in a humidified atmosphere containing 5% CO_2_. Following treatment, samples were processed for RT-qPCR analysis. 

Cell viability of isolated TALs was assessed using 3-(4,5-dimethylthiazol-2-yl)-2,5-diphenyltetrazolium bromide (MTT) assay. TAL samples were incubated with MTT reagent at a final concentration of 0.5 mg/ml for 3–4 h at 37°C in a humidified atmosphere containing 5% CO₂. After incubation, the supernatant was removed, and resulting formazan crystals were dissolved in dimethyl sulfoxide (DMSO). Samples were mixed thoroughly, and absorbance was measured at 570 nm using a microplate reader. Background absorbance was subtracted using the appropriate blank controls. Cell viability was expressed relative to NTC-treated control samples, which were set to 100%.

### ASO synthesis

Standard phosphoramidite synthesis conditions were employed for synthesis of all ASOs on MerMade 96E or MerMade 12 synthesizers (BioAutomation Inc) using 500 Å UnyLinker Controlled Pore Glass (CPG) (ChemGenes). A solution of 3% (V/V) dichloroacetic acid (Sigma–Aldrich) in dichloromethane (DCM) was used for deprotection of dimethoxytrityl (DMT) protecting group. 2′-O-MOE Phosphoramidites (Thermo Fisher Scientific) and DNA phosphoramidites (Thermo Fisher Scientific) were prepared at 0.1 M in extra dry acetonitrile (Thermo Fisher Scientific). A 0.24 M solution of 5-(benzylthio)-1*H*-tetrazole (BTT) (Carbosynth) in dry acetonitrile was used for in-line activation of the phosphoramidites during coupling. Sulfurization replaced the oxidation step using a 0.1 M solution of 3-((*N,N*-dimethylaminomethylidene)amino)-3*H*-1,2,4-dithiazole-5-thione (DDTT) (ChemGenes), in pyridine. 10% (V/V) acetic anhydride in tetrahydrofuran (THF) was used as a Cap A reagent, with 16% N-methylimidazole (Cap B) as a catalyst both (Sigma–Aldrich). The synthesizer cycle parameters used were: Deblock 2 × 60 s; Coupling 2 × 180 s; Sulfurization 1 × 300 s; Capping 2 × 50 s. Cleavage from the solid support CPG was carried out in a 30% ammonium hydroxide solution (Sigma–Aldrich), at 55°C, for 18 h. Following evaporation of ammonia, the ASO was first purified by DMT-on reverse-phase HPLC (Agilent 1200 Series) using an XBridge OST C18 column (2.5 µm, 10 × 50 mm; Waters 186003954) at 65°C, with a flow rate of 5 ml min^−1^ and a 5-min gradient of 23%–60% methanol in 0.1 M aqueous triethylammonium acetate, pH 8.0. The DMT group was then cleaved by treatment with 40% acetic acid for 10 min, followed by a second reverse-phase HPLC purification under otherwise identical conditions, but at 50°C and with a 15%–40% methanol gradient, to remove the liberated DMT.

### ASO transfection

The mIMCD-3 cells were seeded in 24-well plates at a density sufficient to achieve 25%–35% confluency on the day prior to transfection, yielding ~70% confluency at the time of transfection. On the day of transfection, cells were washed once with sterile 1× phosphate buffered saline and cultured in 450 μl of antibiotic-free DMEM. To prepare the ASO-lipoplex, ASOs were first diluted in Opti-MEM (Gibco) and combined with Lipofectamine 2000 (Thermo Fisher Scientific), according to the manufacturer’s instructions, to 10× concentration in 50 μl. The mixture was gently mixed and incubated for 15 min at room temperature to allow complex formation. It was then added directly onto cells in 450 μl fresh medium. The 24-well plates were gently swirled to distribute the complex evenly, and cells were returned to standard culture conditions (37°C, 5% CO_2_) for 48 or 72 h. No antibiotics were present in the culture medium throughout the transfection period.

### *In vitro* RNase H1 assay

Synthetic 47-nt RNA substrates corresponding to the mA6 target region in murine *Umod*-WT and *Umod*-R186S transcripts were prepared and 3′-Cyanine-labeled. Cleavage reactions with mA6 and recombinant human RNase H1 were monitored over time by LC-MS, following a protocol adapted from Kiełpiński *et al*. [25]. Recombinant human GFP-tagged RNase H1 was produced from *Escherichia coli* using the Plasmid #174447 (Addgene) and purified on Ni-NTA agarose (Qiagen) column using the His_6_ tag. The cleavage assays were carried out with 5 µM RNA target strand (2.5 + 2.5 µM in competition experiments) and 2.5 µM ASO, using 0.03 µg/μl RNase H1, in 20 mM Tris–HCl, pH 7.5, 20 mM KCl, 20 mM β-mercaptoethanol, 2 mM MgCl_2_, and 0.1 mM ethylenediaminetetraacetic acid (EDTA). Aliquots were quenched using 50 mM EDTA and then injected on reverse-phase liquid chromatography-coupled mass spectrometry (LC-MS) 1200 series and 6130 quadrupole (both Agilent Technologies) using a gradient of MeOH in buffer: 0.4 M HFIP, 15 mM TEA, of 10%–80% over 10 min. The molecular mass of full-length, Cy-labeled RNA, and fragments, was confirmed using the Oligowizard version 4.2.0 (Oligowizard Ltd.), Oligo Calculator.

### RNA extraction and RT-qPCR

Total RNA was extracted using TRIzol Reagent (Life Technologies) and purified with Zymo-Spin IC Columns (Zymo Research). RNA concentration was measured by NanoDrop. Reverse transcription was performed with the TaqMan™ MicroRNA Reverse Transcription Kit (Applied Biosystems). qPCR was carried out using the KAPA SYBR FAST qPCR Kit (Roche) on a LightCycler 480 (Roche), with primer annealing at 60°C. Primer sequences are listed in (Supplementary Table S2). For allele-specific qPCR, primers were designed with specificity toward WT or R186S cDNA, and validated to exhibit <1% amplification of the off-target allele.

### Mouse models

The *in vivo* protocols were performed on age‐ and sex‐matched *Umod*  ^+/+^ and *Umod*  ^R186S/+^ littermates generated on C57BL/6 background by PolyGene Transgenetics (Rümlang, Switzerland). The *Umod*  ^R186S/+^ mice were previously established as a robust and representative model of the clinical and biological features of ADTKD-*UMOD* [13]. The mice were housed at the laboratory animal service center of the University of Zurich under specific pathogen‐free conditions and maintained under controlled temperature and humidity, with dark/light cycle and *ad libitum* access to tap water and standard chow (Diet AO3, SAFE). Animal procedures were carried out according to protocols approved by the Swiss Cantonal Veterinary Authority (approval number ZH199/20).

### *In vivo* antisense oligonucleotide treatment

Four-week-old mice were treated with an ASO targeting *Umod* (mA6), an NTC ASO, or saline. For the 10-day treatment regimen, mice received three mA6 subcutaneous (*s.c*.) injections of either 50 or 100 mg kg^−1^ in the neck region on days 1, 4, and 8 for a cumulative dose of 150 or 300 mg kg^−1^. Mice were euthanized on day 11, and tissues were collected for subsequent analyses. For the 4-week treatment regimen, treatment was initiated in 4-week-old *Umod*  ^+/+^ and *Umod*  ^R186S/+^ littermates. Mice received three loading doses of mA6 (3 × 50 mg kg^−1^) by intraperitoneal (*i.p*.) injection, followed by seven doses of mA6 (7 × 50 mg kg^−1^) administered *s.c*. in the neck region over the 4-week treatment period. Mice were euthanized on day 32, and tissues were collected for subsequent analyses.

### Protein sample preparation and immunoblot analysis

Kidney tissues and cells were lysed in ice-cold radioimmunoprecipitation assay buffer (Sigma–Aldrich, St.-Louis, MO, USA) supplemented with protease inhibitors (1836153001, Roche, Basel, CH) and phosphatase inhibitors (04906845001, Sigma–Aldrich) using an IKA T10 basic tissue homogenizer (IKA, Staufen, Germany) or cell scraper (99002, TPP Techno Plastic Products, Trasadingen, Switzerland). Lysates were sonicated (amplitude 40%, 1 s, twice) and centrifuged 15 min, 1000 × *g* at 4°C to remove debris. Protein lysates were diluted in 4× Laemmli sample buffer (Bio-Rad, Hercules, CA, USA) and DTT (except for uromodulin), boiled 5 min at 95°C, and loaded. Proteins were separated on sodium dodecyl sulphate–polyacrylamide gel electrophoresis gel and blotted onto PVDF membranes. Membranes were blocked in 5% w/v non-fat dry milk solution and incubated with primary antibodies: anti-human uromodulin (1:500, Invitrogen, PA5-46959) for cells, anti-mouse uromodulin (1:500, Invitrogen, PA5-47706), anti-mouse β-actin (1:10 000, Sigma–Aldrich, A5441), and anti-goat Lcn2 (1:500, R and D Systems, AF1857. Membranes were incubated with peroxidase-conjugated secondary antibodies and visualized by Immobilon Western HRP Substrate (Millipore, Burlington, MA, USA). Immunoblots were quantified by densitometric analysis in ImageJ, with band intensities from cell and kidney samples normalized to β-actin.

### Biochemical analysis and tissue collection

Plasma and urine levels of creatinine, electrolytes, uric acid, and urea were measured on UniCel® DxC 800 Synchron® Clinical System or AU480 Clinical Chemistry System (Beckman Coulter, Brea, CA, USA). Urine and plasma osmolality were measured on a multi-sample osmometer (Advanced Instruments Osmometer 2020, Norwood, MA, USA). The urinary levels of uromodulin were measured with a mouse ELISA Kit (abcam, ab245726) and normalized on urinary creatinine. Following the collection of plasma samples, multiple organs, including kidneys were collected in Clarity OTX Lysis-Loading Buffer v2.0 (Phenomenex). Kidneys were decapsulated; half kidney was flash-frozen for RNA extraction, half was used for SplintR-ligation qPCR analyses, whereas the contralateral kidney was processed for protein extraction (half) and histological (half) analyses.

### Microdissection of TAL and proximal tubule segments

Kidney proximal tubules (PT) and TAL segments were prepared from mouse kidneys briefly digested with Liberase (Roche, Basel, Switzerland), with manual isolation of the segments according to morphologic criteria and validation using specific markers, as described [6, 24]. The samples were lysed in Clarity OTX Lysis-Loading Buffer v2.0 (Phenomenex). Quantitative SplintR-ligation qPCR was performed on pools of ~50–70 tubules for each isolated fraction.

### Quantification of ASOs by SplintR ligation qPCR

Isolated tissues (kidney, liver, spleen, pancreas, heart, and urine) were flash-frozen in liquid N2 after excision and kept at −80°C until processing; TAL cells and PT cells were prepared as described earlier. Due to variation in tissue toughness, harsh lysis conditions were employed for all tissue types. Organ tissues were roughly crushed into pieces of ~20–70 mg, weighed, and added to a pre-labeled 2 ml round-bottom, safe-lock microcentrifuge tube containing one 5 mm stainless steel bead (Sigma–Aldrich). 10 vol/wt Clarity OTX lysis-loading buffer (Phenomenax) was added to the tissue, followed by homogenization in a bead mill (TissueLyser II, Qiagen) at 25 Hz for 4 min. The tissue homogenate was then exposed to freeze-thaw cycling (3X) by submersion in liquid N_2_ followed by 50°C water bath. Homogenization on the bead mill was then repeated, at 25 Hz for 4 min, followed by sonication (bath) at 37 kHz, 30°C for 2 min. The resulting lysed tissue homogenates were diluted for downstream processing and ASO quantification. Absolute quantification of the ASO mA6 in tissue lysates was performed using a ligation-mediated qPCR assay adapted from Shin *et al*. (2022) [26], with optimization based on Boos *et al*. (2013) [27]. Two DNA probes (Microsynth), each partially complementary to adjacent regions of mA6 and containing 5′-phosphate and 3′-hydroxyl termini (Fig. 3 and Supplementary Table S2), were annealed directly to the ASO in a 1:75 dilution of tissue lysate. Hybridization and ligation were carried out by incubating 2 μl of diluted sample with 10 μl of probe mix (2 μl each of 5 µM Probe A and B, 2 μl 10× reaction buffer, and 4 μl ultrapure water) and then adding 8 μl of diluted SplintR Ligase (New England Biolabs) for 2.5 U per reaction, followed by incubation at 30 °C for 30 min followed by 65 °C for 20 min. The ligation product was then amplified via qPCR using Q5 polymerase (New England Biolabs) with Cy3-labeled forward and reverse primers and a BHQ-labeled probe (both Microsynth) (Supplementary Table S2). Each 40 μl qPCR reaction consisted of 33.33 μl of master mix (7 μl 5× Q5 buffer, 0.35 μl Q5 polymerase, 0.35 μl dNTP mix, 1.75 μl each of 10 µM forward and reverse primers, 1.51 μl of 28.28 µM BHQ primer, and 22.05 μl ultrapure water), combined with 6.66 μl of ligated sample. Reactions were run in technical triplicate on a 384-well plate using a LightCycler 480 System (Roche) with the following program: initial denaturation: 95°C for 30 s, 40 cycles of amplification: 95°C 3 s, 50°C for 30 s, and 72°C for 10 s. Quantification was performed by comparison to a calibration curve generated in parallel using known concentrations of mA6 spiked into control tissue lysates from saline-treated mice—treated the same as ASO-treated samples—with mA6 ranging from 250 nM to 0.25 pM. Identical dilutions in ultrapure water were prepared to confirm lysate-specific matrix effects. The mA6 amount was normalized to weight of organ tissue and urine (assuming density: 1 g ml^−1^), or to cell number, through calibration of 18S gDNA. The assay selectively detects intact ASOs with a detection limit of <10 fg (1.4*10^−18^ mole) in water.

### Histological analysis and immunostaining

Kidneys were fixed overnight at 4 °C in 4% formaldehyde (Sigma–Aldrich), dehydrated, and subsequently embedded in paraffin. Paraffin blocks were cut into 5 µm-thick sections, deparaffinized in xylene, and rehydrated in decreasing ethanol concentrations. The sections were incubated with sheep anti-uromodulin (1:300, Invitrogen, PA5-47706), rabbit anti-GRP78 (1:300, Abcam, ab21685), rat Ly-6B (1:400, Abcam, ab53457), rabbit anti-F4/80 (1:200, Cell Signaling, 70076S), and rabbit anti-CD45 (1:100, Cell Signaling, 70257S) overnight at 4°C. After washing steps, sections were incubated with the appropriate Alexa Fluor–labeled secondary antibody (1:400, Life Technologies, Carlsbad, CA) for 1 h at room temperature. The sections were counterstained with 4′,6-diamidino-2-phenylindole (DAPI). Monolayers of mIMCD-3 cells were fixed for 10 min in 4% formaldehyde (Sigma–Aldrich), permeabilized for 30 min using 0.5% saponin (Sigma–Aldrich), and counterstained with Hoechst (Chemie Brunschwig, BIO40047) for 10 min at room temperature. After the last washing step, cells were mounted on a glass slide using Prolong Gold Anti-fade reagent (Invitrogen Corp., Waltham, MA). Kidney slides and cells were viewed under a confocal microscope (Leica Microsystems GmbH, Wetzlar, Germany) using a × 63 1.4 NA oil immersion objective. Quantitative image analysis was performed by randomly selecting five visual fields pooled from biological replicates. The quantitative analyses were performed using ImageJ. Mean intensity of Cy3 or EGFP signals was measured per individual kidney tubule (≥30 tubules) or cell (≥50 cells per condition).

### Colocalization of uromodulin and Cy3 in kidney cross-sections

Fluorescence images were processed in ImageJ/Fiji 2.16.0 to assess colocalization between the Cy3-labeled mA6 probe and apical uromodulin in kidney cortical sections. For each image, binary masks were applied to exclude regions not expressing uromodulin, thereby restricting analysis to the TAL segment. Contrast was manually enhanced for both Cy3 and uromodulin channels to remove background noise and retain only the most intense signal front. Colocalization was then assessed by calculating Pearson’s correlation coefficient (R) between the two channels, reflecting the relative spatial separation between Cy3 and uromodulin signals at 1 and 24 h post-injection.

### Urine and blood collection in mice

Urine samples from mice were collected overnight using individual metabolic cages (UNO Roestvastaal BV, Zevenaar, Netherlands). Blood was collected by venous puncture at time of sacrifice and centrifuged at 2000 × *g* for 15 min at 4°C in heparin-coated tubes (Sarstedt AG, Nürnbrecht, Germany) to separate plasma from cells. BUN (respectively UREA), ALT, and AST levels were measured on the UniCel® DxC 800 Synchron® Clinical System or AU480 Clinical Chemistry System (Beckman Coulter, Brea, CA, USA).

### Statistical analysis

All statistical analyses were performed using GraphPad Prism version 10.4.1. Comparisons between two groups were conducted using unpaired two-tailed *t*-tests. For comparisons involving more than two groups, one-way or two-way analysis of variance (ANOVA) were used with multiple comparisons (Dunnett’s or Tukey’s, respectively). For comparison of different inflammation markers between two groups, inside one plot, multiple *t*-tests were used. Correlation analyses were performed using Pearson correlation coefficients, with *P*-values < .05 considered statistically significant.

## Results

### Allele-selective ASO screening against human uromodulin

ASOs were designed against the human c.553C > A mutation (p.R185S) located on exon 3 of *UMOD* (Fig. 1A), corresponding to the c.556C > A mutation (p.R186S) in murine *Umod* (Fig. 1A and Supplementary Fig. S1). This mutation models a severe form of ADTKD-*UMOD* [13]; it causes early ER stress and kidney aggregates in humans and mice, making it a challenging target for ASO intervention. Since no heterozygous cell line expressing both WT and mutant uromodulin is available, for ASO screening we used mouse kidney tubular (mIMCD-3) cell lines that stably express either *UMOD-*WT or *UMOD-*R185S fused to green fluorescent protein (EGFP) (Fig. 1A and Supplementary Fig. S1). These mIMCD-3 cells lack native uromodulin expression, enabling to assess allele-selective silencing of the engineered human construct without interference from native uromodulin.

Despite recent efforts to develop *in silico* methods [28], reliably predicting potent and selective gapmer ASOs remains an open challenge, and empirical screening continues to be the most dependable strategy for identifying effective sequences. We assembled a library of 20 ASOs for a “microwalk” over the mutation site on the *UMOD* transcript (Supplementary Fig. S2), ensuring full coverage of the mismatch position contra the C > A mutation (Fig. 1B and Supplementary Table S1). The ASOs were synthesized as full PS gapmers, comprising a 10-nt DNA region, flanked by 5-nt 2′MOE “wings” (Supplementary Fig. S3). This modification pattern imparts both enhanced nuclease stability and target-binding affinity and is present in several approved ASO drugs [14, 22].

The ASOs were screened in the mIMCD-3 cell lines against both EGFP*-UMOD*-WT and EGFP*-UMOD*-R185S, at low concentrations [17], in order to highlight the range of selective target suppression (Fig. 1C). Five ASOs (hA6, hA12, hA13, hA17, and hA18) demonstrated acceptable activity with promising allele selectivity and were prioritized for dose escalation studies (Fig. 1D). ASOs hA13 and hA17 exhibited mediocre activity against *UMOD-*R185S and no activity against *UMOD*-WT, except at the highest concentration tested (2 µM), whereas hA12 and hA18 ASOs had better activity against *UMOD-*R185S but also repressed the *UMOD*-WT construct. Only hA6 showed concentration-dependent activity and selectivity against the pathogenic *UMOD-*R185S mRNA in the tested dose range (Fig. 1D). Notably, hA6 features a TC mismatch against the WT RNA in the 5′-terminal gap position, consistent with earlier findings that a mismatch in this position of a target mRNA-ASO hybrid promotes efficient discrimination by RNase H1 [29, 30]. To further demonstrate that the inhibitory effects resulted from sequence-dependent target engagement [31], we employed a sequence-scrambled negative control with identical chemical modifications (NTC). In the mIMCD-3 cells, NTC exhibited minor, insignificant *UMOD* repression compared to vehicle treatment, and lesser activity compared to on-target ASOs (Fig. 1C and D). Immunoblotting analyses and fluorescence microscopy confirmed the selective knockdown of uromodulin, whereby hA6 suppressed mutant (R185S) uromodulin protein by 75% compared to treatment with NTC, whereas WT uromodulin levels remained unchanged (Fig. 1E and Supplementary Fig. S4). Taken together, the cell screening revealed hA6 as a selective inhibitor of mutant *UMOD* and its protein product uromodulin in kidney tubular cells.

### Optimization of the *UMOD* ASO sequence for experiments in ADTKD mice

To study the pharmacokinetics (PK) and pharmacodynamics (PD) properties of ASOs in the mouse model of ADTKD-*UMOD*, the anti-human *UMOD* ASO hA6 was modified at three nucleotides to yield mA6, which is complementary to the equivalent murine *Umod* sequence (Fig. 2A and Supplementary Fig. S1). It seemed plausible that switching three nucleotides in the 3′ wing of hA6 might affect its allele selectivity for the murine *Umod*-R186S over the *Umod-*WT mRNAs. Therefore, we adapted an *in vitro* RNase H assay [25], incubating cyanine-labeled RNA substrates corresponding to the target regions of mA6 on murine *Umod*-WT and mutant *Umod*-R186S mRNAs with mA6 and recombinant human RNase H1, and followed cleavage of the target over time using liquid chromatography–mass spectrometry (LC-MS). Indeed, ASO mA6 cleaved the complementary R186S mRNA significantly faster than it cleaved the WT variant (Fig. 2B and C). Selectivity for the *Umod*-R186S target was maintained when the two RNAs were mixed in an equimolar ratio prior to addition of RNase H and mA6 (Fig. 2D), suggesting that mA6 would exhibit allele-selective activity under competitive conditions in heterozygous *Umod*  ^R186S/+^ mice. After 20-min incubation, LC-MS analysis showed that the main site of cleavage of the R186S RNA was opposite to positions 6–7 of mA6, directly proximal to the *Umod* mutation (Fig. 2E and Supplementary Fig. S5), in agreement with previously published RNase H1 cleavage patterns [32]. In contrast, the major site of cleavage in the mismatched *Umod-*WT RNA was shifted toward the center of the ASO gap, again in agreement with RNase H enzymology [33]. Taken together, the data provide a well-precedented mechanistic rationale for the allele-selectivity of mA6 and hA6.

**Figure 2.**
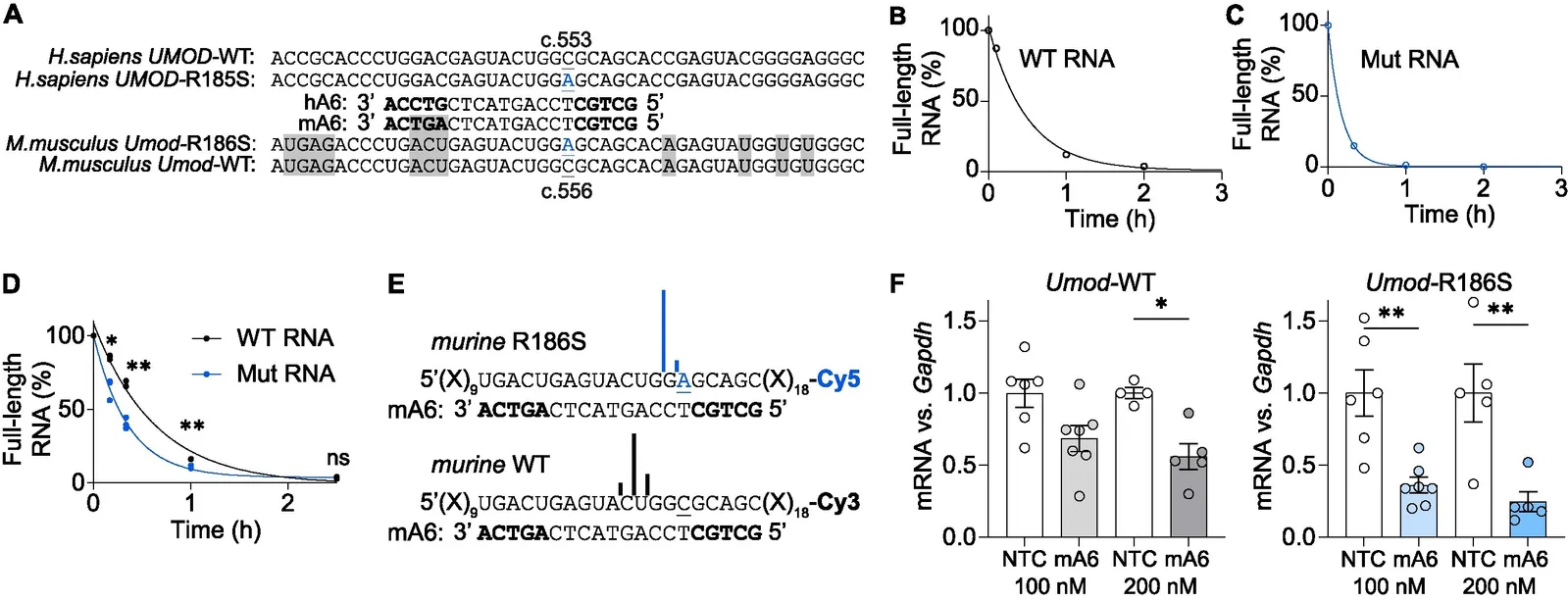
Design and validation of allele-selective murine-targeting ASO mA6. (**A**) Sequence alignment of the murine and human target region surrounding the pathogenic UMOD mutation and binding sites of hA6 and mA6. Murine-specific bases are shaded in gray; the C > A SNP is underlined, and the mutant allele is shown in blue. 2′-MOE modifications are indicated in bold. RNase H1–mediated cleavage of synthetic 47-nt murine WT (**B**) and Mut (**C**) RNA by mA6. Full-length RNA was quantified by LC peak integration at 554 nm (Cy3, WT) and 651 nm (Cy5, Mut) (Supplementary Fig. S5). (**D**) Competitive cleavage of WT (black) and mutant (blue) *Umod* RNA by mA6 (*N* = 3). (**E**) Cleavage site mapping after a 20-min reaction. 3′-fragments were identified by LC–MS and quantified. (**F**) Allele-specific RT-qPCR of WT and mutant *Umod* RNA from isolated TAL (*Umod*  ^R186S/+^) tubules after transfection with mA6 or NTC for 48 h.

Next, we isolated TAL microtubules from *Umod*  ^R186S/+^ kidneys through microdissection. The isolated mRNA showed a robust signal by qPCR for *Umod* and no detectable signal for *Aqp2* and *Lrp2* mRNAs, confirming the purity of the TAL samples (Supplementary Fig. S5). Transfection of mA6 into the microtubules was performed at 100 and 200 nM concentrations, and mRNA was isolated after 48 h. Caspase-3 and MTT assays showed no significant change, indicating that mA6 transfection at these concentrations did not impair tubular cell viability (Supplementary Fig. S5). Using allele-specific qPCR primers for *Umod*-R186S and *Umod*-WT—validated to exhibit <1% off-target amplification—we found that mA6 delivered a strong, concentration-dependent reduction of *Umod*-R186S and only a weak reduction in *Umod*-WT mRNA (Fig. 2F). Other genes characteristically expressed by TAL cells were not affected by ASO treatment, supporting the sequence-dependent engagement of mA6 (Supplementary Fig. S5). The results confirmed that mA6 could provide a selective inhibition of *Umod*-R186S in *Umod*  ^R186S/+^ mouse-derived TAL segments.

### Pilot dosing study of ASO distribution and target engagement in *Umod*  ^R186S/+^ mice

In ADTKD-*UMOD*, tissue damage is driven by aggregation of misfolded uromodulin in TAL cells (Fig. 3A). In contrast to kidneys of *Umod ^+/+^* mice, showing only mature uromodulin (∼100 kDa), kidneys of *Umod*  ^R186S/+^ mice show premature (p-UMOD, ∼80 kDa) and high-molecular-weight (HMW, >200 kDa) uromodulin aggregates. As previously reported [13], disease severity in this ADTKD-*UMOD* model is such that even at 4 weeks of age, the mature isoform of uromodulin is barely detectable (Fig. 3B). Notably, levels of *Umod* mRNA in *Umod*  ^R186S/+^ kidneys are substantially lower than in *Umod*  ^+/+^ kidneys (Fig. 3C), consistent with an uncharacterized feedback mechanism that suppresses *Umod* mRNA upon mutant uromodulin accumulation.

**Figure 3.**
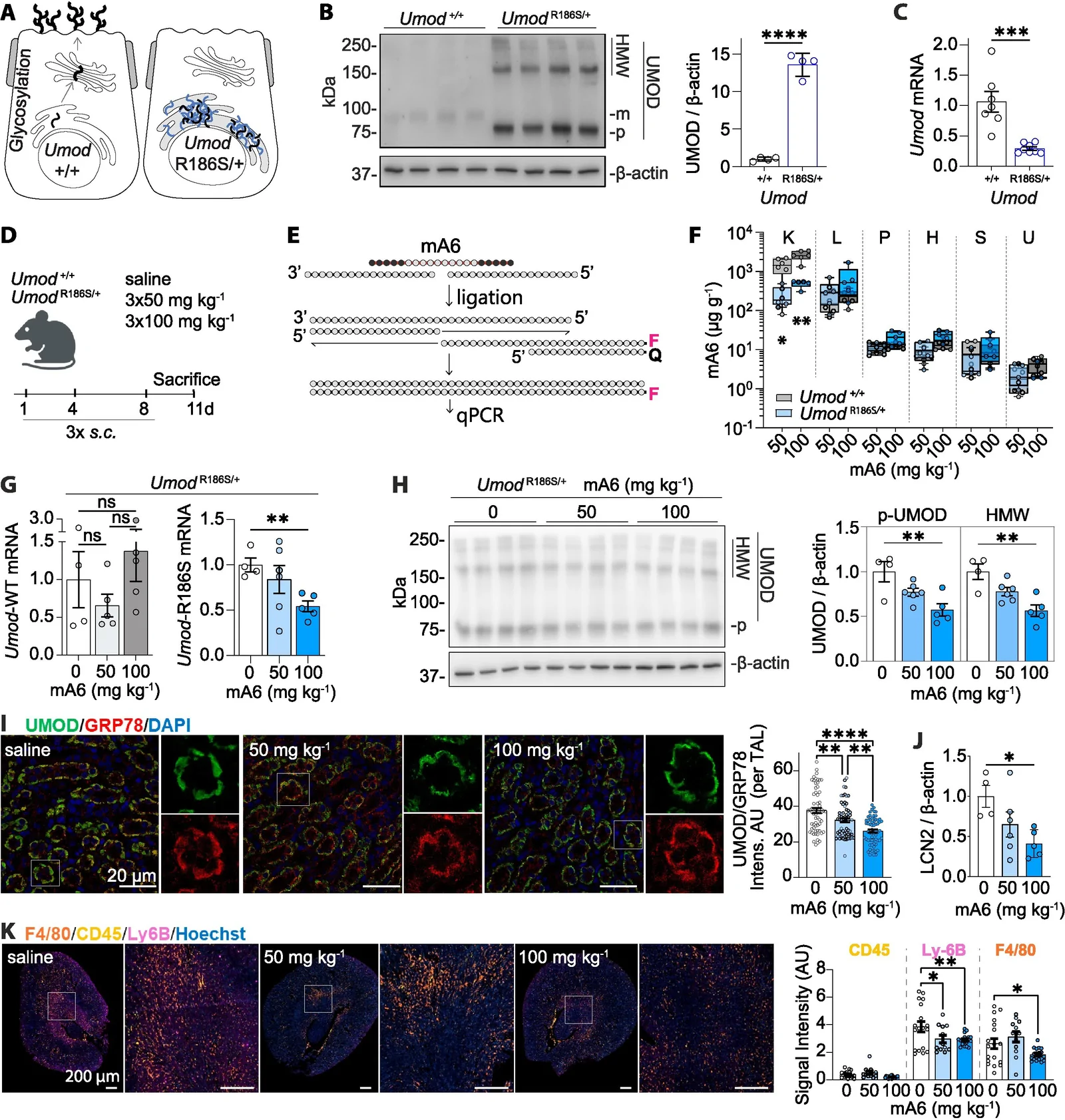
Tissue accumulation and activity of mA6 in pilot study of mouse model of ADTKD-*UMOD*. (**A**) Illustration of the fate of WT and mutant UMOD in TAL cells. (**B**) Immunoblots and their quantification showing the accumulation of UMOD in saline-treated *Umod*  ^+/+^ and *Umod*  ^R186S/+^ mice. Mature (m-UMOD), premature (p-UMOD), and HMW UMOD isoforms. (**C**) Total *Umod* mRNA levels in kidneys of saline-treated *Umod*  ^+/+^ and *Umod*  ^R186S/+^ mice. (**D**) 10-day protocol with three doses of 0 (saline), 50, or 100 mg kg^−1^ mA6 in *Umod*  ^+/+^ and *Umod*  ^R186S/+^ littermates (*N* ≥ 4). (**E**) Principle of SplintR-ligation qPCR for detection of full-length mA6. (**F**) Tissue accumulation of mA6 following 10-day treatment of *Umod*  ^+/+^ and *Umod*  ^R186S/+^ mice (K: kidney; L: liver; P: pancreas; H: heart; S: spleen; U: urine). Box-and-whisker plots show the median (line), interquartile range (box), and range (whiskers). (**G**) Allele-selective qPCR analysis of kidneys of *Umod*  ^R186S/+^ mice, treated with three doses of 0, 50, or 100 mg kg^−1^ mA6, normalized to *Gapdh*. (**H**) Representative western blot and UMOD quantification, normalized to β-actin, from *Umod*  ^R186S/+^ kidney lysates following treatment (see also Supplementary Fig. S6). (**I**) Immunofluorescence staining and colocalization of UMOD and GRP78 in *Umod*  ^R186S/+^ kidneys. (**J**) Levels of the tubular stress marker lipocalin-2 (LCN2) in kidneys after mA6 treatment, quantified by western blot (Supplementary Fig. S6). (**K**) Multiplex immunofluorescence staining and quantification of CD45, Ly6B, and F4/80 in medullary cross-sections of *Umod*  ^R186S/+^ kidneys. Data expressed as means ± SEM; *P < .05, **P < .01, ***P < .001, and ^****^P < .0001 indicate comparisons with *t*-test (**F**), one-way ANOVA with multiple comparisons (G, H, I, J), or two-way ANOVA with multiple comparisons (K).

Although ASOs accumulate in kidneys following systemic administration [34, 35], their productive distribution is limited by the highly differentiated nature of tubular cells [36, 37]. Furthermore, ASO PK can be significantly altered in disease state [17, 38], potentially limiting organ distribution and target engagement. Given these constraints, we carried out a short dose-finding protocol to examine target engagement and biodistribution of mA6 in *Umod*  ^R186S/+^ and *Umod*  ^+/+^ littermates. Mice (4-week-old) were administered *s.c*. three doses of saline or mA6 at 50 or 100 mg kg^−1^ over 10 days (Fig. 3D) (Supplementary Fig. S6 and Supplementary Table S3). We estimated that these doses would promote high kidney uptake within tolerated levels [39], consistent with previous studies using ASOs in kidney disease models [17, 18, 40].

Full-length mA6 was quantified using a dedicated splint-ligation qPCR assay (Fig. 3E) [26]. Mouse samples were fitted to calibration curves, which were established from relevant tissue lysates (Supplementary Fig. S6). Following the 50 and 100 mg kg^−1^ treatments, we measured mA6 in kidneys (∼1400 and ∼2600 µg g^−1^, respectively) and livers (∼180 and ∼240 µg g^−1^, respectively) of *Umod ^+/+^* mice (Fig. 3F). These values were consistent with those reported in studies using different methods [15, 41]. While the overall biodistribution was similar in *Umod*  ^R186S/+^ and *Umod*  ^+/+^ mice, a significantly lower uptake was seen in kidneys of *Umod*  ^R186S/+^ mice, i.e. carrying kidney damage (Fig. 3F).

Mindful of potential feedback between *Umod* mRNA and protein after mA6 treatments (Fig. 3B and C), we measured levels of *Umod* mRNAs in *Umod*  ^R186S/+^ mice by allele-selective qPCR. *Umod*-WT mRNA was not significantly reduced by mA6 treatment at either dose, whereas *Umod*-R186S mRNA was reduced by approximately 50% at the 100 mg kg^−1^ dose (Fig. 3G). Correspondingly, uromodulin was reduced in kidneys of treated *Umod*  ^R186S/+^ mice after three doses of 100 mg kg^−1^ (Fig. 3H) (Supplementary Fig. S6). At this dose, both premature uromodulin (p-UMOD) and HMW uromodulin aggregates were significantly repressed compared with vehicle treatment. In order to confirm that uromodulin suppression was not due to an off-target effect of mA6, we treated a second cohort of mice with three doses of 100 mg kg^−1^ of the NTC ASO. As expected, NTC showed no effects on levels of p-UMOD and HMW-UMOD proteins (Supplementary Fig. S7).

Importantly, we did not have a mechanistic expectation that mA6 would reduce HMW uromodulin aggregate levels below those already present at the start of treatment, therefore mA6 would likely only prevent new aggregates. A robust correlation between p-UMOD and HMW aggregates in kidneys was observed across the three treatment groups, as expected since toxic uromodulin aggregates are generated by polymerization of premature uromodulin (Supplementary Fig. S6).

Target suppression was substantiated by dose-dependent reductions of uromodulin retained in the ER, shown by colocalization with GRP78 in TAL cells (Fig. 3I). In addition, mA6-treated mice exhibited lower lipocalin-2 (LCN2), a marker of tubular injury (Fig. 3J), which also correlated with residual levels of HMW uromodulin (Supplementary Fig. S6). Conversely, treatment with NTC at 100 mg kg^−1^ did not affect GRP78 or LCN2 levels, consistent with its lack of effect on mutant uromodulin species (Supplementary Fig. S7). Moreover, significant reductions in neutrophil (Ly6B) and macrophage (F4/80) infiltrates were detected in mA6-treated *Umod*  ^R186S/+^ kidneys (Fig. 3K). Altogether, the results from the dose-finding experiments revealed that systemically administered ASO achieved a sequence-specific TAL cell targeting and suppression of mutant uromodulin species, with reduced ER retention associated with decreased tubular injury and milder interstitial infiltrates.

### Saline-formulated ASOs enter and accumulate in the nucleus of kidney TAL cells

For an effective ADTKD-*UMOD* therapy, ASOs should enter TAL cells and track to the nucleus. To provide insights on ASO internalization by TAL cells, we labeled mA6 at its 5′ end with cyanine 3 (mA6-Cy3), using a stable PS linkage. The ASO probe was then administered to *Umod*  ^+/+^ and *Umod*  ^R186S/+^ mice via a single intraperitoneal (*i.p*.) injection at 50 mg kg^−1^. The Cy3 conjugate reportedly does not influence the broad organ/tissue distribution of labeled oligonucleotides after systemic administration in mouse [42]; however, we could not definitively rule out that the probe might influence entry into TAL cells. We compared medullary sections from WT and mutant kidneys harvested 1 and 24 h post-administration. At 1 h, the Cy3 signal in TAL cells of *Umod*  ^+/+^ kidneys appeared to be strongest near the basolateral membrane (Fig. 4A), shifting significantly toward the nucleus by 24 h, quantified by the change in spatial separation from apical uromodulin (*R* = −0.88 at 1 h; *R* = −0.34 at 24 h). The *Umod*  ^R186S/+^ kidneys also exhibited Cy3 signals near the basolateral membrane of TAL cells at 1 h, shifting to the nuclei at 24 h (Fig. 4B). Here, the negative correlation was strong at both time points (*R* = −0.96 at 1 h versus *R* = −0.94 at 24 h), possibly due to uromodulin misprocessing and accumulation in the ER. Furthermore, at 1 h the Cy3 signal appeared more diffuse in *Umod*  ^+/+^ compared to *Umod*  ^R186S/+^ kidneys. Together, these data suggested that mA6-Cy3 is taken up into TAL cells via basolateral entry, consistent with systemic distribution, rather than urinary exposure [43, 44]. Since Cy3 is relatively hydrophobic and can alter ASO distribution, the localization data with mA6-Cy3 should be interpreted with caution, as it may not fully represent the unlabeled ASO. More detailed time-course experiments, using appropriate apical and basolateral markers and measuring kinetics of ASO excretion in urine, would be needed in order to draw robust conclusions on the mechanism of cell entry. Importantly, both mouse models demonstrated nuclear accumulation of the ASO within 24 h, consistent with target knockdown.

**Figure 4.**
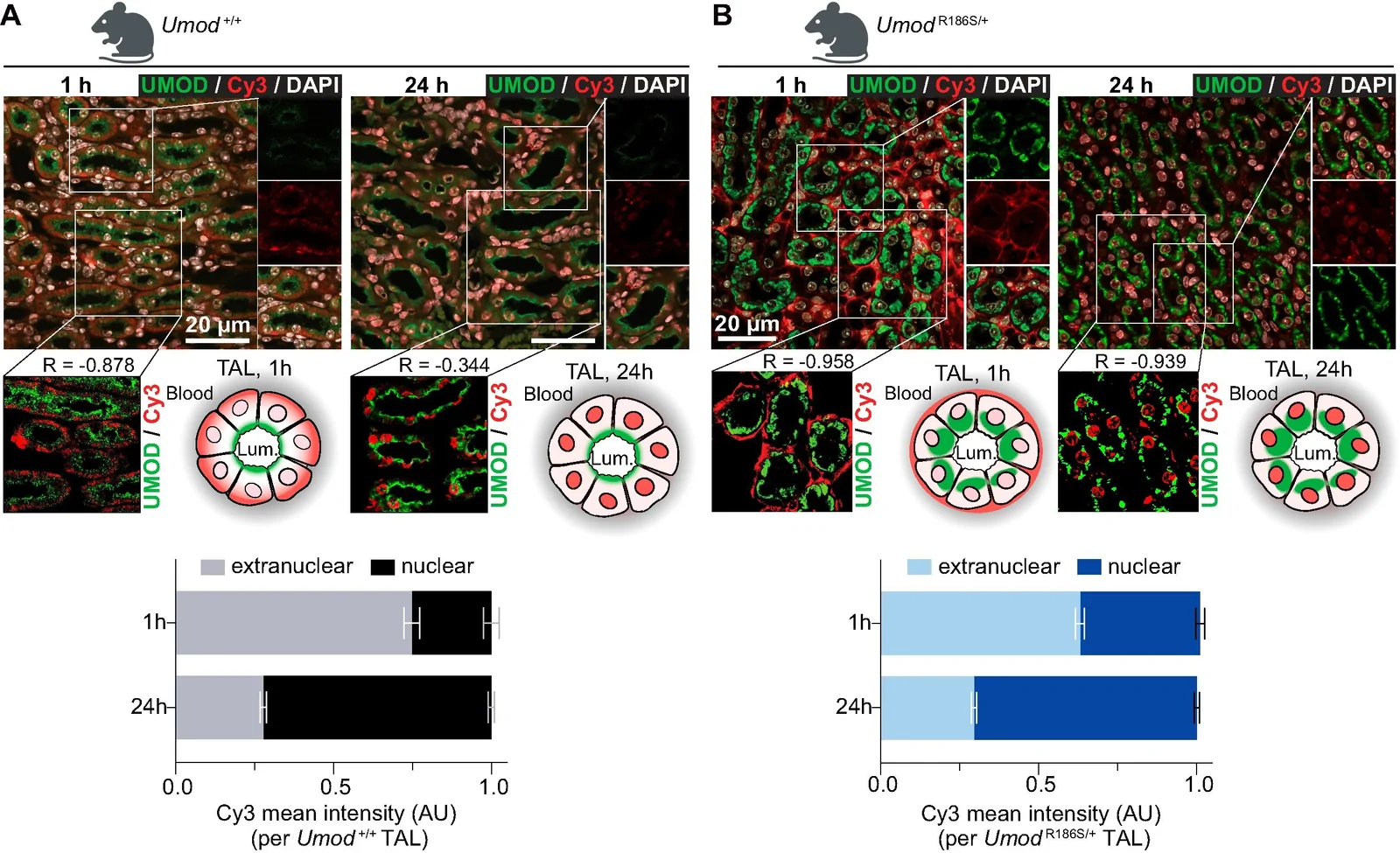
ASO trafficking to cells lining the TAL of Henle’s loop. Intraperitoneal administration of Cy3-labeled mA6 into (**A**) *Umod*  ^+/+^ mouse and (**B**) *Umod*  ^R186S/+^ mouse. (A, B) Representative immunofluorescence analysis 1 and 24 h post-injection of uromodulin (UMOD, green) and Cy3 (red) signals on kidney sections from 4-week old *Umod*  ^+/+^ and *Umod*  ^R186S/+^ mice (*n*  = 2–3 animals per group). Scale bar: 20 μm. Nuclei (gray) are counterstained with DAPI. Enhanced contrast images of TAL cells, isolated with a mask, are shown, with pseudo-quantitative colocalization (correlation coefficient R) of the Cy3 and UMOD signal fronts at both time points. Cartoons show interpretation of signal fronts from the enhanced contrast images. Bar graphs show quantification (mean ± SEM), from original photographs, of extranuclear and nuclear fractions of Cy3 in TAL cells at 1 and 24 h.

### Allele-selective therapy reduces uromodulin aggregates and kidney damage in *Umod*  ^R186S/+^ mice

In view of the positive results obtained with the mA6 and NTC ASOs in the dose-finding pilot study, we modified the protocol to a 4-week regimen employing three loading doses of 50 mg kg^−1^  *i.p*., followed by seven doses of 50 mg kg^−1^  *s.c*. injections in 4 week-old *Umod*  ^+/+^ and *Umod*  ^R186S/+^ littermates (Fig. 5A). With this extended regimen, kidney accumulation of mA6 was similar in *Umod*  ^+/+^ and *Umod*  ^R186S/+^ mice, reaching levels of ∼1000 µg g⁻¹ in mutant mice compared with 250 µg g⁻¹ in the pilot study (Fig. 5B and Supplementary Fig. S8). Increased ASO administrations also enhanced mA6 accumulation in other organs, including the liver and heart (Fig. 5B). The extended dosing regimen did not affect weight gain, appetite or behavior, but induced increases in alanine aminotransferase (ALT) and aspartate aminotransferase (AST) levels in treated *Umod*  ^R186S/+^ and *Umod*  ^+/+^ mice (Supplementary Fig. S9), similar to those reported by other groups working with 2′-MOE gapmer ASOs [45]. Importantly, mA6 treatment did not affect blood urea nitrogen (BUN) or serum creatinine levels (Supplementary Fig. S9 and Supplementary Tables S4 and S5), unlike a previously described kidney-targeted ASO that was terminated for these reasons [46].

**Figure 5.**
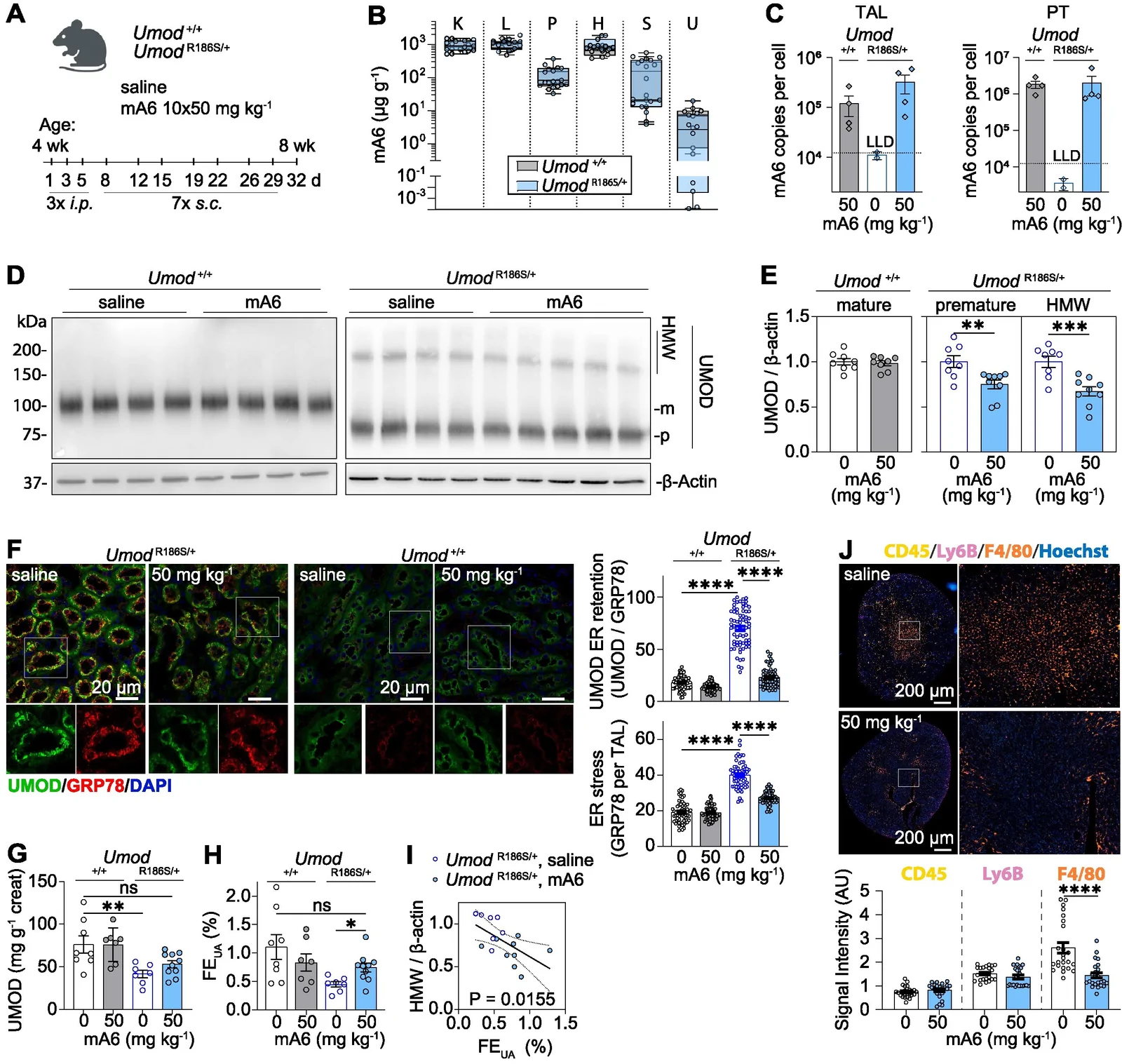
Effects of selective mutant UMOD suppression in *Umod*  ^R186S/+^ mice. (**A**) Four-week protocol with 10 doses of: 0 (saline) or 50 mg kg^−1^ mA6 in *Umod*  ^+/+^ and *Umod*  ^R186S/+^ mice (*N* ≥ 7). (**B**) Tissue accumulation of mA6 following treatment (K: kidney; L: liver; P: pancreas; H: heart; S: spleen; U: urine). (**C**) Absolute accumulation of mA6 molecules per cell in TAL and PT segments (*N* = 4), following treatment. Untreated mouse samples registered below the lower limit of detection (LLD) of the assay. (**D-E**) Representative immunoblots and blot densitometry from kidney lysates of *Umod*  ^+/+^ and *Umod*  ^R186S/+^ following treatment with saline or mA6 (Supplementary Fig. S10). The blot shows mature (m), premature (p) and HMW UMOD forms depending on the genotype. (**F**) Immunofluorescence staining and colocalization of UMOD and the ER stress marker GRP78, in TAL cells and quantification of the signals in *Umod*^+/+^ and *Umod*  ^R186S/+^ kidneys. (**G**) Influence of mA6 treatment on urinary UMOD documented at the treatment endpoint. (**H**) FE_UA_ of *Umod*  ^R186S/+^ and *Umod*  ^+/+^ mice at the treatment endpoint. (**I**) Correlation between FE_UA_ and HMW-UMOD. (**J**) Multiplex immunofluorescence staining and quantification of CD45, Ly6B and F4/80 in medullary kidney cross-sections of *Umod*  ^R186S/+^ mice treated with saline or mA6. Data expressed as mean ± SEM; *P < .05, **P < .01, ***P < .001, and ^****^P < .0001 of t test comparison (**E**) one-way ANOVA (**F, G, H**) and multiple *t*-test comparisons (**J**).

Detailed examination of the ASO accumulation in isolated tubular segments [24], calibrated against 18S gDNA (Supplementary Fig. S8) revealed an average of 320 000 mA6 molecules per cell in *Umod*  ^R186S/+^ and 120 000 molecules per cell in *Umod*  ^+/+^ TAL segments (Fig. 5C). Although we are not aware of any previous studies reporting absolute ASO copy number per cell from tissues or organs *in vivo*, our data aligns with that found for effective gapmer and splice switching activity *in vitro*: ∼100 000 copies per cell for MCF-7 cells [47], 247 000–693 000 copies per nuclei in HeLa cells [48] and 375 000–710 000 copies per cell in HEL cells [49]. Nearly 2 × 10^6^ copies of mA6 per cell were found in PT segments (Fig. 5C), commensurate with reports of ASO uptake into this cell type [47, 50]. Since ~1% of systemically administered ASOs are reportedly cleared via the urine within 24 h of administration [51, 52], we collected urine throughout the treatment and determined mA6 elimination. ASO levels in urine increased exponentially over the treatment course, reaching 0.6% of the last dose at the treatment endpoint, with no observed difference between *Umod*  ^+/+^ and *Umod*  ^R186S/+^ mice (Supplementary Fig. S8).

Because mA6 contains a mismatch to the WT allele that impairs target engagement, the treatment of *Umod*  ^+/+^ mice served as a negative control for the experiments, matching ASO chemistry, sequence, and exposure with no target engagement. The 4-week treatment with 50 mg kg^−1^ mA6 had no effect on uromodulin levels in *Umod*  ^+/+^ mice (Fig. 5D-E). Conversely, in the context of uromodulin levels at treatment onset (Supplementary Fig. S10), the mA6 treatment significantly inhibited formation of new pathogenic HMW uromodulin aggregates in *Umod*  ^R186S/+^ mice (Fig. 5D-E), confirming its allele-selective activity. The significant effect of mA6 on formation of HMW uromodulin aggregates correlated with the effect on p-UMOD in these kidneys (Supplementary Fig. S8). In line, the mA6 treatment robustly reduced ER-retained uromodulin in TAL cells to levels comparable to WT mice (Fig. 5F), paralleled by significant reductions in ER stress levels as indicated by reduced signal for GRP78 in TAL cells (Fig. 5F) and lower *Hspa5* mRNA levels in treated kidneys (Supplementary Fig. S8).

The effect of mA6-mediated reductions of mutant uromodulin aggregates in treated kidneys was further assessed on several clinically relevant outcomes. As expected, urinalysis showed higher levels of baseline uromodulin secretion in vehicle-treated *Umod*  ^+/+^ mice compared to *Umod*  ^R186S/+^ mice (Supplementary Tables S4 and S5). The mA6-treatment narrowed this gap in *Umod*  ^R186S/+^ mice, but the change did not reach significance (Fig. 5G). The lack of correlation between urinary uromodulin levels and HMW uromodulin aggregates (Supplementary Fig. S8) may indicate that urinary uromodulin level was a poor predictor for the degree of HMW repression in this context. Conversely, the fractional excretion of uric acid (FE_UA_), which is typically reduced in patients with ADTKD-*UMOD* [53], was significantly increased by mA6 ASO-treatment in *Umod*  ^R186S/+^ mice, approaching levels seen in WT mice (Fig. 5H). Whereas mA6 treatment of *Umod*  ^+/+^ mice showed no change upon mA6 treatment, a significant negative correlation between FE_UA_ and levels of HMW uromodulin aggregates hinted at a beneficial effect of reducing the toxic aggregates on tubular function (Fig. 5I). Furthermore, mA6 treatment was reflected by a significant rescue of macrophage (F4/80) infiltration in *Umod*  ^R186S/+^ kidneys, compared to saline treated littermates (Fig. 5J). Taken together, these data indicated that uromodulin suppression with mA6 in the *Umod*  ^R186S/+^ mice was associated with improved markers of tubular damage and milder immune reaction, substantiating the translational potential of allele-selective ASO treatment in ADTKD-*UMOD*.

## Discussion

Oligonucleotide drugs are well-positioned to address toxic gain-of-function disorders and are the only class of synthetic drugs presently able to target disease-linked mRNAs with allele-selectivity [20, 54]. Much of the recent success in liver targeting is attributed to a tri-antennary GalNAc ligand, which, when conjugated to small interfering RNAs (siRNAs) and ASOs, ensures an efficient, receptor-mediated uptake into hepatocytes [55]. Intensive research in industry and academia is underway to identify alternative targeting ligands [56], and exciting progress has been reported for delivery to specific areas of the brain [57], the skeletal muscle [58], and the pancreas [59]. Paradoxically, oligonucleotide development has not advanced past clinical trials for the kidney, the organ that typically shows the highest accumulation of an unformulated oligonucleotide after systemic administration [15]. This may be at least partly due to the complexity of highly differentiated cells operating in various nephron segments and their selective vascular compartments, which renders cell entry difficult for oligonucleotides [37]. Nevertheless, pre-clinical and clinical research is advancing for polycystic kidney disease [17, 18], for Alport syndrome [40], and for type 2 diabetes [46].

ADTKD is one of the most common types of genetic kidney diseases. Missense mutations in *UMOD* cause the majority of diagnosed cases of ADTKD, triggering defects in uromodulin processing and formation of toxic intracellular aggregates [1, 2]. There are no current therapeutic options for ADTKD, although one recent report describes a small molecule targeting transmembrane Emp24 protein transport domain-containing (TMED) cargo receptors to rescue uromodulin trafficking, with no effect reported on aggregation [60]. The adult-onset and slowly progressive nature of ADTKD-*UMOD* offer a therapeutic window, while the link between uromodulin misprocessing and disease severity suggests that even incomplete rescue could help alleviating phenotype [61, 62].

Strategies for targeting *UMOD* in the context of ASO therapy can follow two distinct approaches: selectively targeting the mutant allele or suppressing *UMOD* expression altogether. Although deletion of WT uromodulin does not cause overt adverse effects in mice [63], its presence in ADTKD-*UMOD* mouse models limits aggregate formation and slows disease progression [13]. This suggests that while a pan-*UMOD* targeting approach could be feasible, a selective approach may be advantageous in preventing aggregation while maintaining physiological functions of uromodulin, including defense against urinary tract infections [8]. For these reasons, and considering that consequences of biallelic *UMOD* knock-out in humans are unknown, we have chosen to pursue an allele-selective approach. Accordingly, we developed PS MOE gapmer ASOs targeting the severe c.553C > A (p.R185S) *UMOD* mutation as a proof-of-concept for this allele-selective approach. Follow-up studies building on this work could include testing ASOs that reduce expression of both WT and mutant *UMOD* alleles, also in less severe, slowly progressive disease models.  

Screening PS-MOE gapmers covering the mutation yielded a lead ASO that featured a mismatch to WT *UMOD* in the 5′-gap position, fully consistent with reports of favorable discrimination by RNase-H1 at this site [29, 30]. We confirmed the importance of the mismatch to allele selectivity using an *in vitro* RNase H assay. The ASO was subsequently shown to target selectively *Umod*  ^R186S/+^ mRNA and protein in ADTKD tubules and mutant kidneys, whereas a scrambled NTC sequence displayed no such effects, confirming the on-target activity of mA6 *in vivo*. The ASO mA6 had no measurable effects on WT protein in WT mice. We did not optimize the ASO further, but structural modifications would be expected to improve potency. For instance, shortening or lengthening the gap region, substituting 2′-*O*-methyl groups into the gap [64], adding locked-nucleic acids residues to the wings and exchanging selected PS groups for PO groups [65, 66] have all been shown to improve potency, selectivity and toxicity of ASOs. In addition, a combination of steps focused on improving potency and TAL-delivery would likely relieve some of the moderate activation of liver ALT and AST levels observed with our lead ASO and enable a reduction in dosing frequency.

Reports of ASO delivery to specific organ cell types *in vivo* are rare. In this study, we quantified ASO uptake into the targeted TAL segment using a dedicated, sensitive splint ligation assay. We also provided preliminary insights on the direction of ASO trafficking into polarized epithelial cells using a fluorophore-labeled ASO. This latter approach would be of importance for approaches that relied upon targeted apical or basolateral delivery via selectively expressed receptors. Our data are most consistent with uptake occurring from the basolateral membrane, which contrasts with the only other study reporting use of ASOs in TAL cells, posited that uptake occurred over the apical membrane, and only after ischemic injury [67]. While we cannot fully exclude the possibility of apical entry, our observations support basolateral access as a relevant route under the conditions examined here.

Importantly, our findings showed that mA6 ASO treatment could silence mutant uromodulin, in a severe aggregation-prone model of ADTKD-*UMOD* and consistently reverse the phenotypic effects specifically observed in *Umod*  ^R186S/+^ mice as compared to their *Umod*  ^+/+^ WT littermates, including both kidney tubular function and inflammation markers. Hence, treatment of *Umod*  ^+/+^ mice with the same ASO did not suppress WT uromodulin, nor alter any of these markers, consistent with the on-target activity of the ASO in the disease model. It is possible that ASO effects might be enhanced in less severe ADTKD models, e.g. those harboring the C125R or C171Y mutations, characterized by a lower propensity to form toxic uromodulin aggregates in TAL cells [13, 68]. Future ASO against TAL-specific toxic gain-of-function protein could aim at expanding the strategy to the wider patient population [23].

While this study provides proof-of-concept for allele-selective ASO therapy in ADTKD-*UMOD*, a number of limitations should be acknowledged. First, we focused on a single *UMOD* mutation (R185S), and additional variants may differ in their response to ASO-mediated silencing. Second, ASO uptake was reduced in diseased kidneys at early time points; while this was partially mitigated by increasing dose and treatment duration, impaired delivery in compromised tissue may remain a challenge for translation. Finally, elevated ALT/AST levels observed at the therapeutic dose highlight the need for more potent ASOs with improved tissue specificity, particularly given the likelihood of chronic administration in a clinical setting. Nevertheless, these results offer a roadmap for antisense therapy to treat ADTKD-*UMOD* and expand the field of ASO application beyond liver hepatocytes into an organ of high medical interest.

## Supplementary Material

gkag1007_Supplemental_File

## Data Availability

All data supporting the findings of this study are available within the article and its Supplementary Data. Raw data underlying the results are available from the corresponding authors upon reasonable request.
