# Supplementary material for: Allele-selective targeting of uromodulin by antisense oligonucleotides reduces aggregates and kidney damage in a mouse model of autosomal dominant tubulointerstitial kidney disease
Source: Nucleic Acids Res. 2026 Nov 10;54(21):gkag1007. doi: 10.1093/nar/gkag1007 (PMC13748981; doi:10.1093/nar/gkag1007)
Supplement: Supplementary file 1 [file gkag1007_supplemental_file.pdf]

Supplementary Material

Allele-selective targeting of uromodulin by antisense oligonucleotides reduces aggregates and kidney damage in a mouse model of autosomal dominant tubulointerstitial kidney disease

Sebastian Sjöström†, Marta Mariniello†, Guglielmo Schiano, Jennifer Lake, Sara Botta, Alyssa Hill, Jonathan Hall#*, Olivier Devuyst#*

Corresponding authors: Olivier Devuyst, olivier.devuyst@uzh.ch; Jonathan Hall, jonathan.hall@pharma.ethz.ch

The PDF file includes:
Figs. S1 to S10; Tables S1 to S5

Table of Contents

Fig. S1. Target human and mouse uromodulin sequences. 2

Fig. S2. Human EGFP-*UMOD* cDNA sequence in mIMCD-3 cells. 3

Fig. S3. Analytics of purified ASOs by liquid chromatography. 4

Fig. S4. hA6-mediated suppression of EGFP-*UMOD* expression in mIMCD-3 cells. 5

Fig. S5. Characterization of mA6 sequence-specific activity against murine RNA.......... 6

Fig. S6. Pharmacokinetics and therapeutic effect of 10-day mA6-treatment in *Umod* $^{+/+}$ and *Umod* $^{R186S/+}$ mice. 7

Fig. S7. Lack of activity of NTC ASO in *Umod* $^{R186S/+}$ kidneys.......... 8

Fig. S8. Pharmacokinetics and therapeutic effect of 4-week mA6-treatment in the *Umod* $^{+/+}$ and *Umod* $^{R186S/+}$ mice. 9

Fig. S9. Safety and tolerability of mA6 in *Umod* mouse models. 10

Fig. S10. Progression of UMOD accumulation and effect of mA6-treatment in *Umod* $^{+/+}$ and *Umod* $^{R186S/+}$ mouse kidneys.......... 11

Table S1. Analytics of purified ASOs by mass spectrometry. 12

Table S2. DNA primers and probes.......... 13

Table S3. Clinical and biological parameters for *Umod* $^{R186S/+}$ mice after 10-day treatment. 14

Table S4. Clinical and biological parameters for *Umod* $^{R186S/+}$ mice after 4-week treatment. 15

Table S5. Clinical and biological parameters for *Umod* $^{+/+}$ mice after 4-week treatment.......... 16

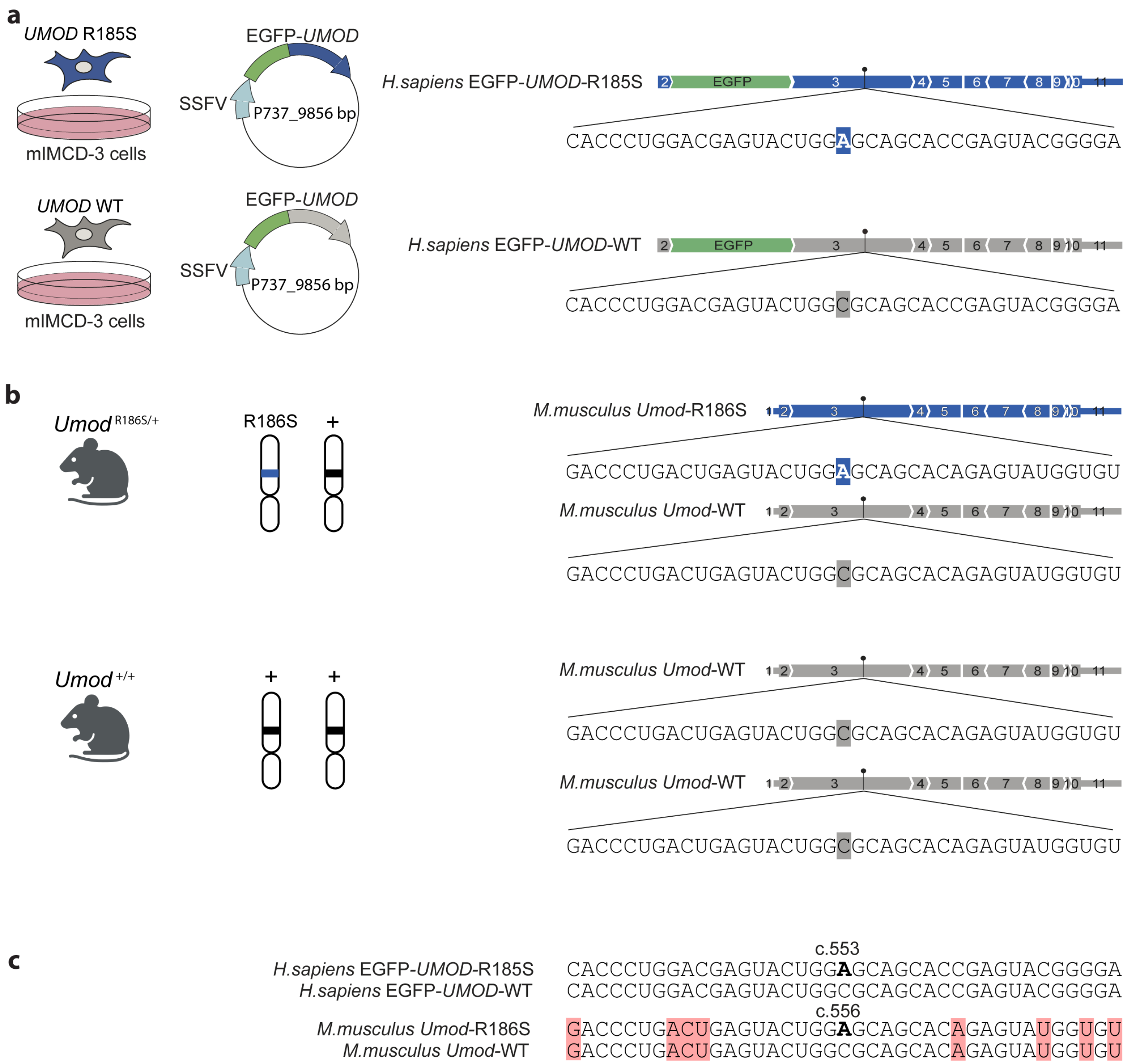

Fig. S1. Target human and mouse uromodulin sequences. a) mIMCD-3 cells engineered to express human *UMOD* fused with EGFP: EGFP-*UMOD*-R185S or EGFP-*UMOD*-WT. A c.553C>A point mutation (black dot and line) on exon 3, distinguishes the pathological (R185S) mutant (blue) from the wild-type (grey). The sequence of the 39 nt ASO-library target region is given showing c.553A and wild-type c.553C bases highlighted. **b)** Heterozygous *Umod* $^{R186S/+}$ mouse model of ADTKD-*UMOD* and wild-type *Umod* $^{+/+}$, with position of the corresponding mutation, c.556. **c)** Comparison of the human and mouse sequences (**a, b**); the divergent bases in mouse are highlighted in red. The pathological mutant sites c.553A and c.556A are shown in bold. Exon maps were generated with https://exonviz.rnatherapy.nl/ using NCBI Reference Sequence: NM_001008389.3 (*H. sapiens*), and NM_001278605.1 (*M. musculus*).

ATGGGGCAGCCATCTCTGACTTGGATGCTGATGGTGGTGGTGGCCTCTTGGTTCATCACAACTGCAGCCACTGA
CACCGGTGGTATGGTGAGCAAGGGCGAGGAGCTGTTCACCGGGGTGGTGCCCATCCTGGTCGAGCTGGACGGCG
ACGTAAACGGCCACAAGTTCAGCGTGTCCGGCGAGGGCGAGGGCGATGCCACCTACGGCAAGCTGACCCTGAAG
TTCATCTGCACCACCGGCAAGCTGCCCGTGCCCTGGCCCACCCTCGTGACCACCCTGACCTACGGCGTGCAGTG
CTTCAGCCGCTACCCCGACCACATGAAGCAGCACGACTTCTTCAAGTCCGCCATGCCCGAAGGCTACGTCCAGG
AGCGCACCATCTTCTTCAAGGACGACGGCAACTACAAGACCCGCGCCGAGGTGAAGTTCGAGGGCGACACCCTG
GTGAACCGCATCGAGCTGAAGGGCATCGACTTCAAGGAGGACGGCAACATCCTGGGGCACAAGCTGGAGTACAA
CTACAACAGCCACAACGTCTATATCATGGCCGACAAGCAGAAGAACGGCATCAAGGTGAACTTCAAGATCCGCC
ACAACATCGAGGACGGCAGCGTGCAGCTCGCCGACCACTACCAGCAGAACACCCCCATCGGCGACGGCCCCGTG
CTGCTGCCCGACAACCACTACCTGAGCACCCAGTCCGCCCTGAGCAAAGACCCCAACGAGAAGCGCGATCACAT
GGTCCTGCTGGAGTTCGTGACCGCCGCCGGGATCACTCTCGGCATGGACGAGCTGTACAAGACCGGTTCAGAAG
CAAGATGGTGCTCTGAATGTCACAGCAATGCCACCTGCACGGAGGATGAGGCCGTTACGACGTGCACCTGTCAG
GAGGGCTTCACCGGCGATGGCCTGACCTGCGTGGACCTGGATGAGTGCGCCATTCCTGGAGCTCACAACTGCTC
CGCCAACAGCAGCTGCGTAAACACGCCAGGCTCCTTCTCCTGCGTCTGCCCCGAAGGCTTCCGCCTGTCGCCCG
GTCTCGGCTGCACAGACGTGGATGAGTGCGCTGAGCCTGGGCTTAGCCACTGCCACGCCCTGGCCACATGTGTC
AATGTGGTGGGCAGCTACTTGTGCGTATGCCCCGCGGGCTACCGGGGGGATGGATGGCACTGTGAGTGCTCCCC
GGGCTCCTGCGGGCCGGGGTTGGACTGCGTGCCCGAGGGCGACGCGCTCGTGTGCGCGGATCCGTGTCAGGCGC
ACCGCACCCTGGACGAGTACTGG<u>**A**</u>GCAGCACCGAGTACGGGGAGGGCTACGCCTGCGACACGGACCTGCGCGGC
TGGTACCGCTTCGTGGGCCAGGGCGGTGCGCGCATGGCCGAGACCTGCGTGCCAGTCCTGCGCTGCAACACGGC
CGCCCCCATGTGGCTCAATGGCACGCATCCGTCCAGCGACGAGGGCATCGTGAGCCGCAAGGCCTGCGCGCACT
GGAGCGGCCACTGCTGCCTGTGGGATGCGTCCGTCCAGGTGAAGGCCTGTGCCGGCGGCTACTACGTCTACAAC
CTGACAGCGCCCCCCGAGTGTCACCTGGCGTACTGCACAGACCCCAGCTCCGTGGAGGGGACGTGTGAGGAGTG
CAGTATAGACGAGGACTGCAAATCGAATAATGGCAGATGGCACTGCCAGTGCAAACAGGACTTCAACATCACTG
ATATCTCCCTCCTGGAGCACAGGCTGGAATGTGGGGCCAATGACATGAAGGTGTCGCTGGGCAAGTGCCAGCTG
AAGAGTCTGGGCTTCGACAAGGTCTTCATGTACCTGAGTGACAGCCGGTGCTCGGGCTTCAATGACAGAGACAA
CCGGGACTGGGTGTCTGTAGTGACCCCAGCCCGGGATGGCCCCTGTGGGACAGTGTTGACGAGGAATGAAACCC
ATGCCACTTACAGCAACACCCTCTACCTGGCAGATGAGATCATCATCCGTGACCTCAACATCAAAATCAACTTT
GCATGCTCCTACCCCCTGGACATGAAAGTCAGCCTGAAGACCGCCCTACAGCCAATGGTCAGTGCTCTAAACAT
CAGAGTGGGCGGGACCGGCATGTTCACCGTGCGGATGGCGCTCTTCCAGACCCCTTCCTACACGCAGCCCTACC
AAGGCTCCTCCGTGACACTGTCCACTGAGGCTTTTCTCTATGTGGGCACCATGTTGGATGGGGGCGACCTGTCC
CGATTTGCACTGCTCATGACCAACTGCTATGCCACACCCAGTAGCAATGCCACGGACCCCCTGAAGTACTTCAT
CATCCAGGACAGATGCCCACACACTAGAGACTCAACTATCCAAGTGGTGGAGAATGGGGAGTCCTCCCAGGGCC
GATTTTCCGTCCAGATGTTCCGGTTTGCTGGAAACTATGACCTAGTCTACCTGGACTGTGAAGTCTATCTCTGT
GACACCATGAATGAAAAGTGCAAGCCTACCTGCTCTGGGACCAGATTCCGAAGTGGGAGTGTCATAGATCAATC
CCGTGTCCTGAACTTGGGTCCCATCACACGGAAAGGTGTCCAGGCCACAGTCTCAAGGGCTTTTAGCAGCTTGG
GGCTCCTGAAAGTCTGGCTGCCTCTGCTTCTCTCGGCCACCTTGACCCTGACTTTTCAGTGA

Fig. S2. Human EGFP-*UMOD* cDNA sequence in mIMCD-3 cells. The sequence of EGFP-*UMOD*-R185S inserted into the P737__9856_bp plasmid. The start- and stop codons are indicated with grey highlighting. The *EGFP* sequence is shown in green, inserted between exons 2-3. The mutation c.553C>A is shown in bold and underlined. The light blue sequence indicates an AgeI restriction site, which was inserted between positions 78 and 79 of the *UMOD* cDNA, immediately after the region encoding the signal peptide. This sequence was used to design the human *UMOD*-targeting ASO library in (**Fig. 1**).

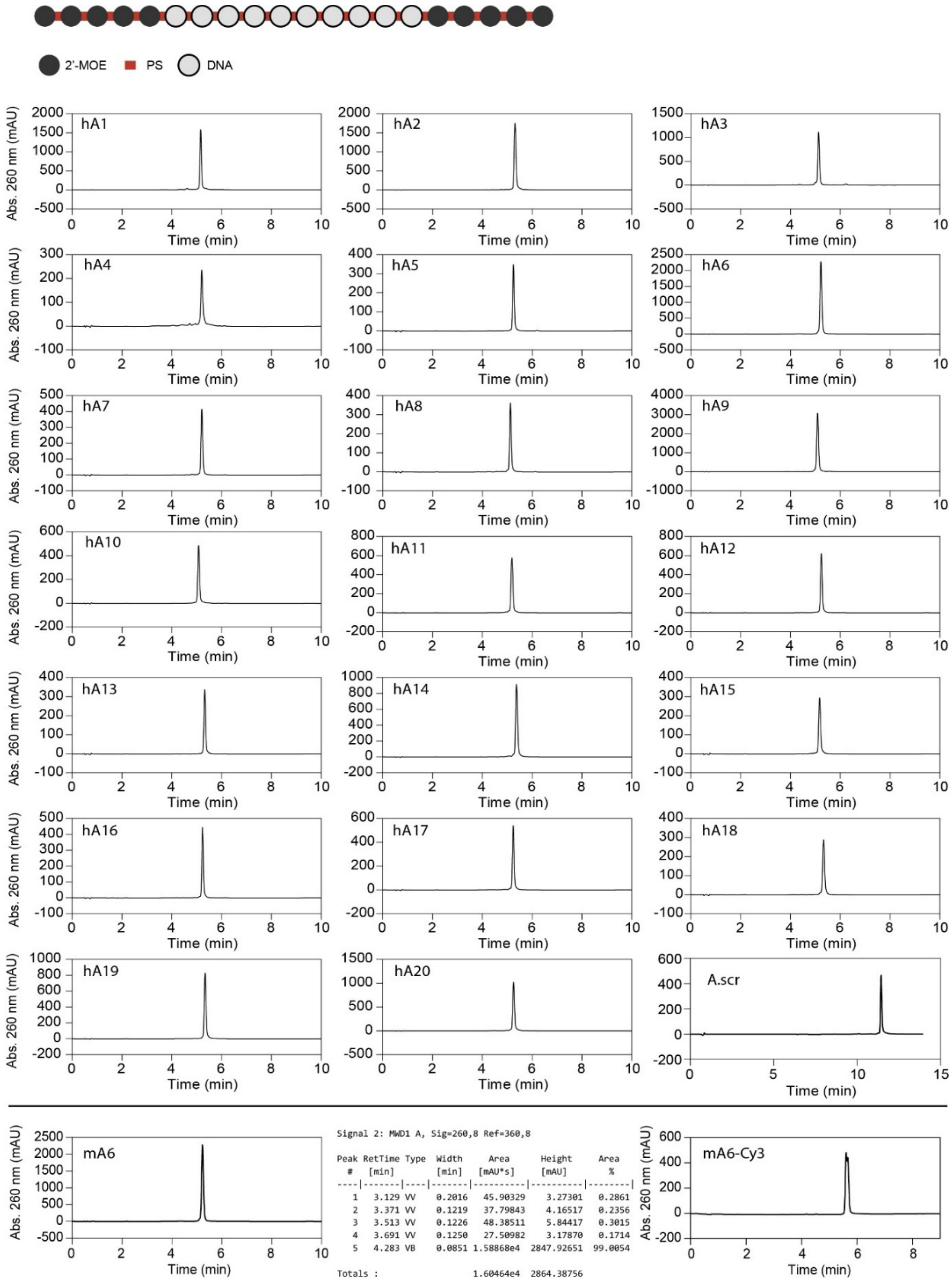

Peak #	RetTime [min]	Type	Width [min]	Area [mAU*s]	Height [mAU]	Area %
1	3.129	VV	0.2016	45.90329	3.27301	0.2861
2	3.371	VV	0.1219	37.79843	4.16517	0.2356
3	3.513	VV	0.1226	48.38511	5.84417	0.3015
4	3.691	VV	0.1250	27.50982	3.17870	0.1714
5	4.283	VB	0.0851	1.58868e4	2847.92651	99.0054
Totals :				1.60464e4	2864.38756	

Fig. S3. Analytics of purified ASOs by liquid chromatography. Illustration showing the chemical modification pattern of PS-DNA/MOE gapmer ASOs, and chromatogram of twice HPLC-purified ASOs targeting the pathogenic human c.553C>A mutation. Chromatogram collected with a MeOH gradient in HFIP (0.4 M), TEAA (14 mM) of 5-80%, 0.3 mL/min, using abs: 260 nm, with reference wavelength 360 nm. Coupled mass spectrometry analysis validating the ASO identity is shown in (**Table S1**). The mA6 purity (99%) was determined by automatic integration of UV chromatogram peaks.

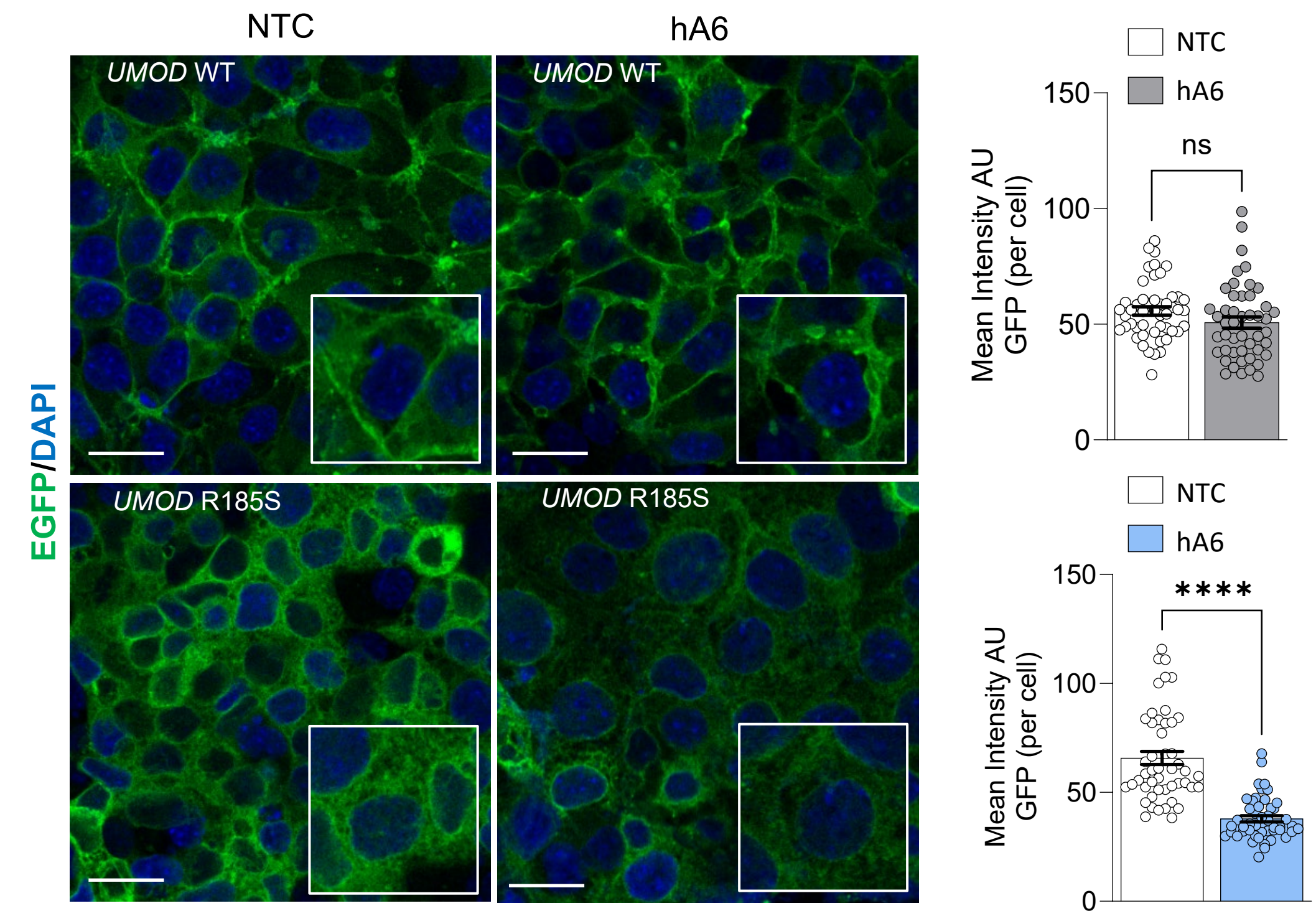

Fig. S4. hA6-mediated suppression of EGFP-*UMOD* expression in mIMCD-3 cells. Representative immunofluorescence analysis of EGFP signal in mIMCD-3 cells expressing either wild-type (WT) or mutant (R185S) human uromodulin. White squares indicate images shown at higher magnification. Scale bar: 30 µm and 15 µm. Cells were transfected with 1.6 µM hA6 or a scrambled control (NTC) and incubated for 72 h. Nuclei were counterstained with DAPI. Data are presented as mean fluorescence intensity ± SEM. Statistical significance was determined using a two-tailed Student's t-test; ****P < 0.0001 versus NTC control.

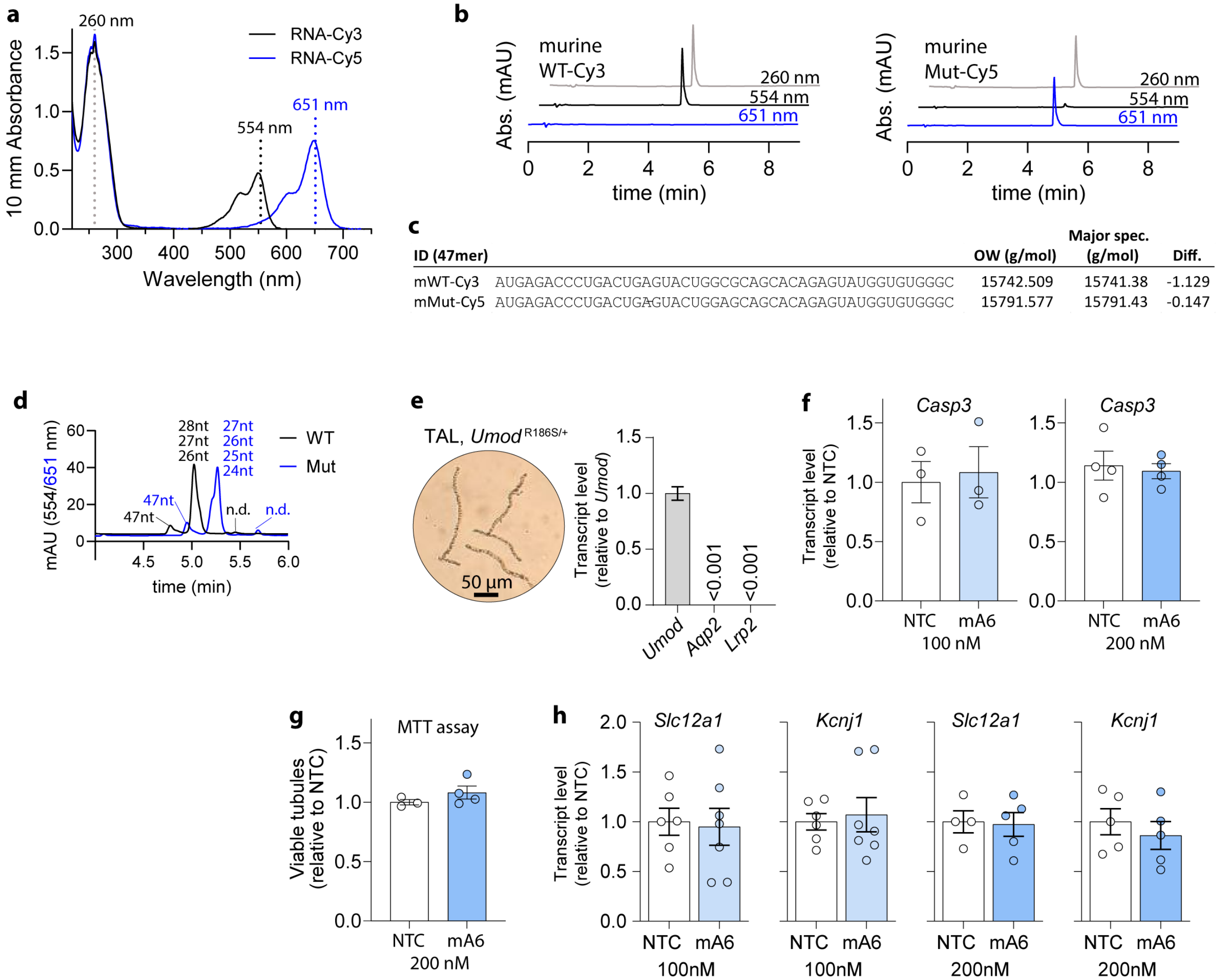

Fig. S5. Characterization of mA6 sequence-specific activity against murine RNA. a) Absorbance spectra of synthetic 47 nt RNA – corresponding to the mA6 target region – labeled at the 3'-terminus with Cy3 (WT) or Cy5 (Mut). The maximum absorbance wavelengths of the dyes are indicated. **b)** LC-MS chromatograms of cyanine-labeled murine RNAs, measured at 260 nm, 554 nm, and 651 nm. **c)** Sequences and molecular weight, calculated for the Cy-labeled RNA using oligowizard.com (OW). **d)** LC-MS chromatogram of cleavage fragments after 20 min of incubation of the RNA with RNase H1 and mA6. The fragments were identified by matching their molecular weights to theoretically possible 3'-cleavage products (OW). **e)** Microscopy picture of isolated murine TAL segments; identity and purity of the TAL segments confirmed by RT-qPCR for *Umod* (TAL), *Aqp2* (DCT) and *Lrp2* (PT). **f)** qPCR analysis of caspase-3 (*Casp3*) expression in isolated TALs, transfected with mA6 at 100 nM or 200 nM. Expression levels are shown as fold change ($2^{-\Delta\Delta Ct}$) relative to the non-targeting control (NTC). **g)** MTT assay measuring viable tubules in isolated TAL segments transfected with mA6 at 200 nM, shown relative to NTC. **h)** Quantification of NKCC2 (*slc12a1*) and ROMK (*Kcnj1*) mRNA levels by RT-qPCR, normalized to NTC treatment.

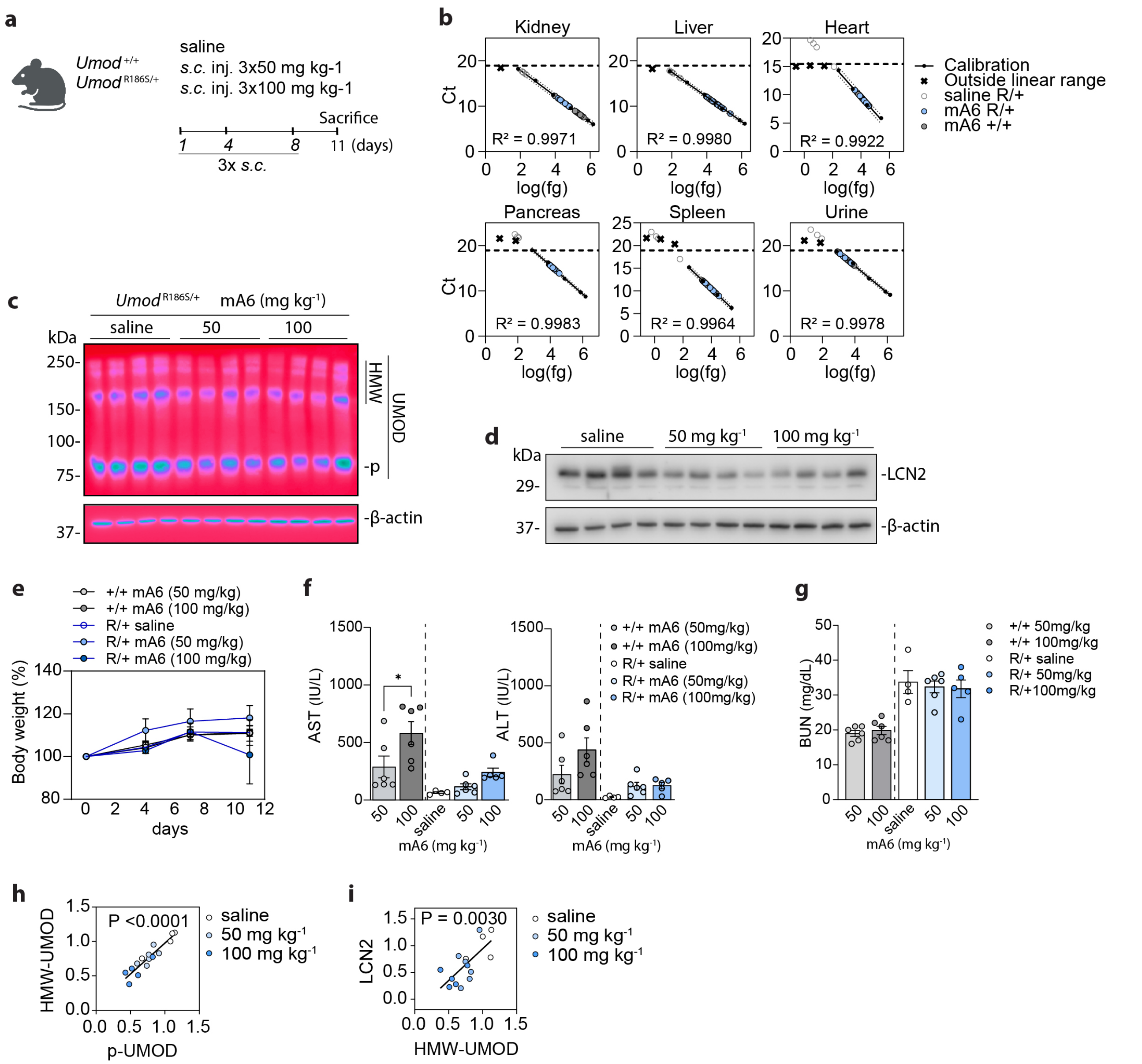

Fig. S6. Pharmacokinetics and therapeutic effect of 10-day mA6-treatment in *Umod*$^{+/+}$ and *Umod*$^{R186S/+}$ mice. a) Protocol with three 50 mg kg^{-1} *s.c.* administration of mA6 over ten days. **b)** Calibration curves by splintR ligation qPCR, created by spiking known amounts of mA6 into tissue lysates of saline-treated littermates, and carrying out the assay on these lysates alongside the treated mouse samples. Values from mA6-treated mice are interpolated into calibration curves. Dashed line represents the Ct value measured by spiking in water into the lysate, and values above the line fall below the detection limit. **c)** Western blot from (**Fig. 3H**) shown using a false-color LUT (Ice, ImageJ) representation of pixel densities, without modification of the original data. **d)** Immunoblot for lipocalin-2 (LCN2) and β-actin from kidney lysates from *Umod*$^{R186S/+}$ mice following 10-day treatment. **e)** Body weight gain throughout intervention, expressed as % of starting body weight. **f)** Circulating levels of aspartate aminotransferase (AST) and alanine aminotransferase (ALT) measured in blood at the 10-day treatment endpoint. **g)** Blood urea nitrogen (BUN) levels in mice at 10-day endpoint blood collection. **h)** Correlation between premature p-UMOD and HMW-UMOD protein levels in kidneys of *Umod*$^{R186S/+}$ mice after treatment. **i)** Correlation between HMW-UMOD and LCN2 protein levels in kidneys of *Umod*$^{R186S/+}$ mice after treatment. Data are presented as the mean of biological replicates ± SEM. *$P<0.05$ indicate one-way ANOVA comparisons with multiple comparisons Dunnett's post hoc (n = 4-6 animals).

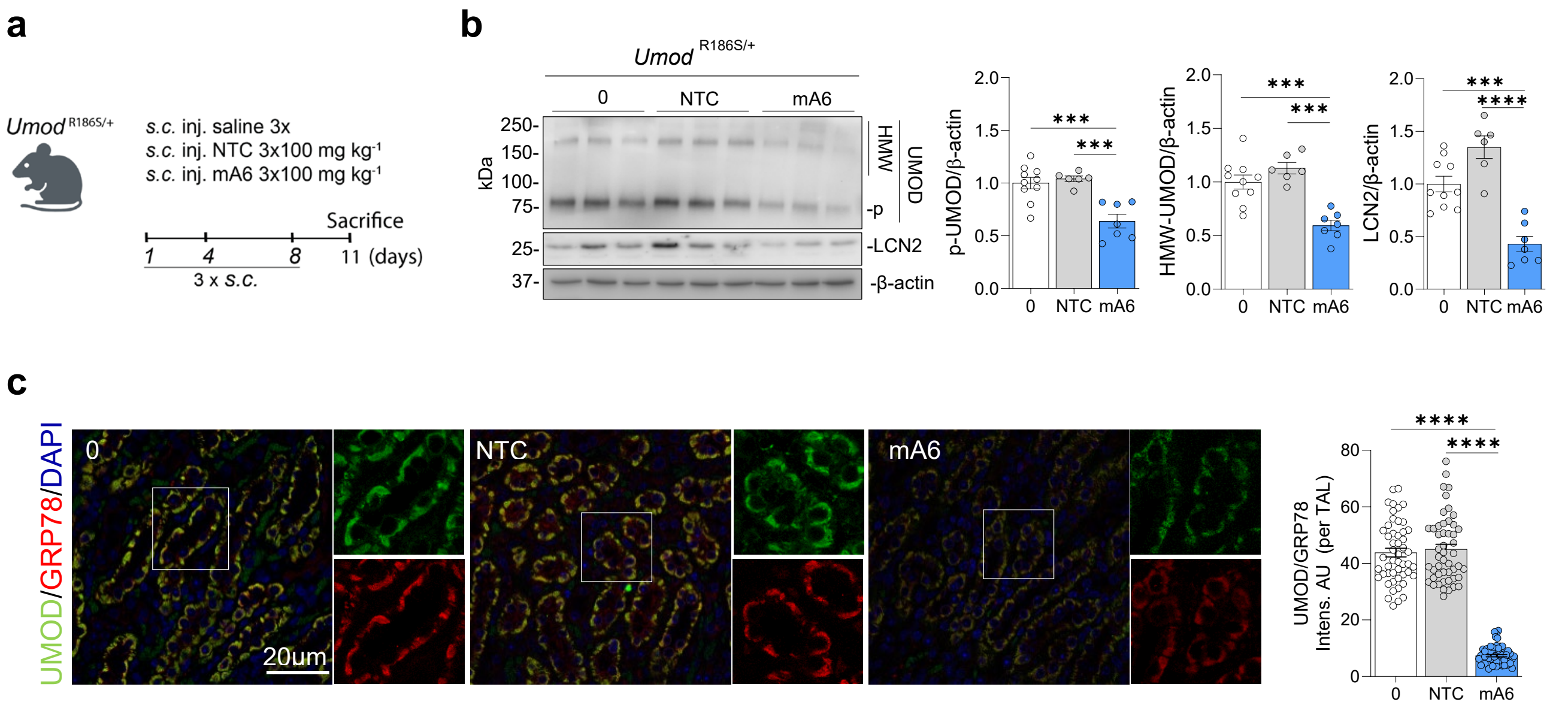

Fig. S7. Lack of activity of NTC ASO in *Umod*$^{R186S/+}$ kidneys. a) Experimental scheme for *Umod*$^{R186S/+}$ mice treated with three *s.c.* injections of saline (0), NTC (100 mg kg⁻¹), or mA6 ASO (100 mg kg⁻¹) on days 1, 4, and 8, followed by sacrifice on day 11. **b)** Representative western blots and corresponding quantification of p-UMOD, HMW-UMOD, and LCN2 in kidney lysates. β-actin was used as a loading control. Densitometry analyses were performed across independent blots (saline, *n* = 10; NTC, *n* = 6; mA6, *n* = 7). **c)** Representative immunofluorescence images of kidney sections stained for UMOD (green) and GRP78 (red), with nuclei counterstained with DAPI (blue). Scale bar, 20 µm. Boxed regions are shown at higher magnification. Data are presented as mean ± SEM. Statistical significance assessed by one-way ANOVA with Tukey's multiple-comparisons test or by Kruskal–Wallis test (**b**, **c**). ***$P < 0.001$, and ****$P < 0.0001$.

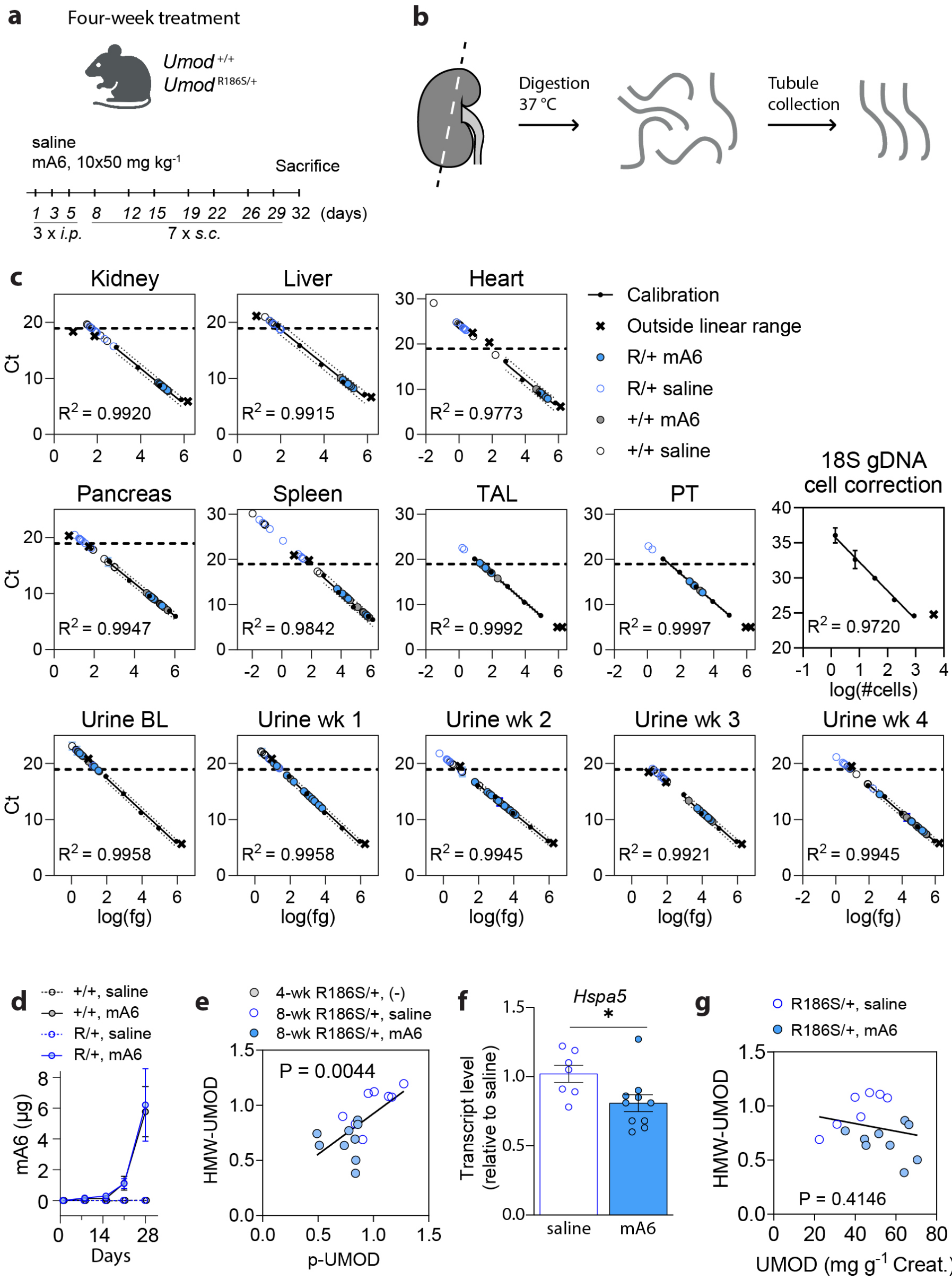

Fig. S8. Pharmacokinetics and therapeutic effect of 4-week mA6-treatment in the *Umod*$^{+/+}$ and *Umod*$^{R186S/+}$ mice. a) Protocol with three *i.p.* injections 50 mg kg^{-1} and seven *s.c.* injections 50 mg kg^{-1} mA6 over four weeks in *Umod*$^{+/+}$ and *Umod*$^{R186S/+}$ mice. **b)** Isolation of kidney tubules through microdissection and digestion, for subsequent quantification of mA6 by SplintR ligation qPCR. **c)** Calibration curves by splintR ligation qPCR, created by spiking known amounts of mA6 into tissue lysates of saline-treated littermates and carrying out the assay on these lysates alongside the treated mouse samples. Values from mA6-treated mice are interpolated into the calibration curves. Dashed line represents the Ct value measured by spiking in water into the lysate, and values above the line fall below the detection limit. Liver and kidney lysates were diluted 1:10 with respect to other lysates, to interpolate them within the calibration curve. **d)** Urinary ASO excretion over the 4-week treatment. **e)** Correlation between the premature p-UMOD and HMW-UMOD protein levels in kidneys of *Umod*$^{R186S/+}$ mice treated for 4 weeks. **f)** Effect of treatment on mRNA levels of *Hspa5* (GRP78), measured by RT-qPCR and normalized to *Gapdh*. **g)** Correlation between urinary uromodulin and HMW-UMOD protein levels in kidneys of mice treated for 4 weeks. Data are expressed as mean ± SEM. Statistical significance was determined using a two-tailed Student's t-test (n = 7–10 animals per group). *P<0.05.

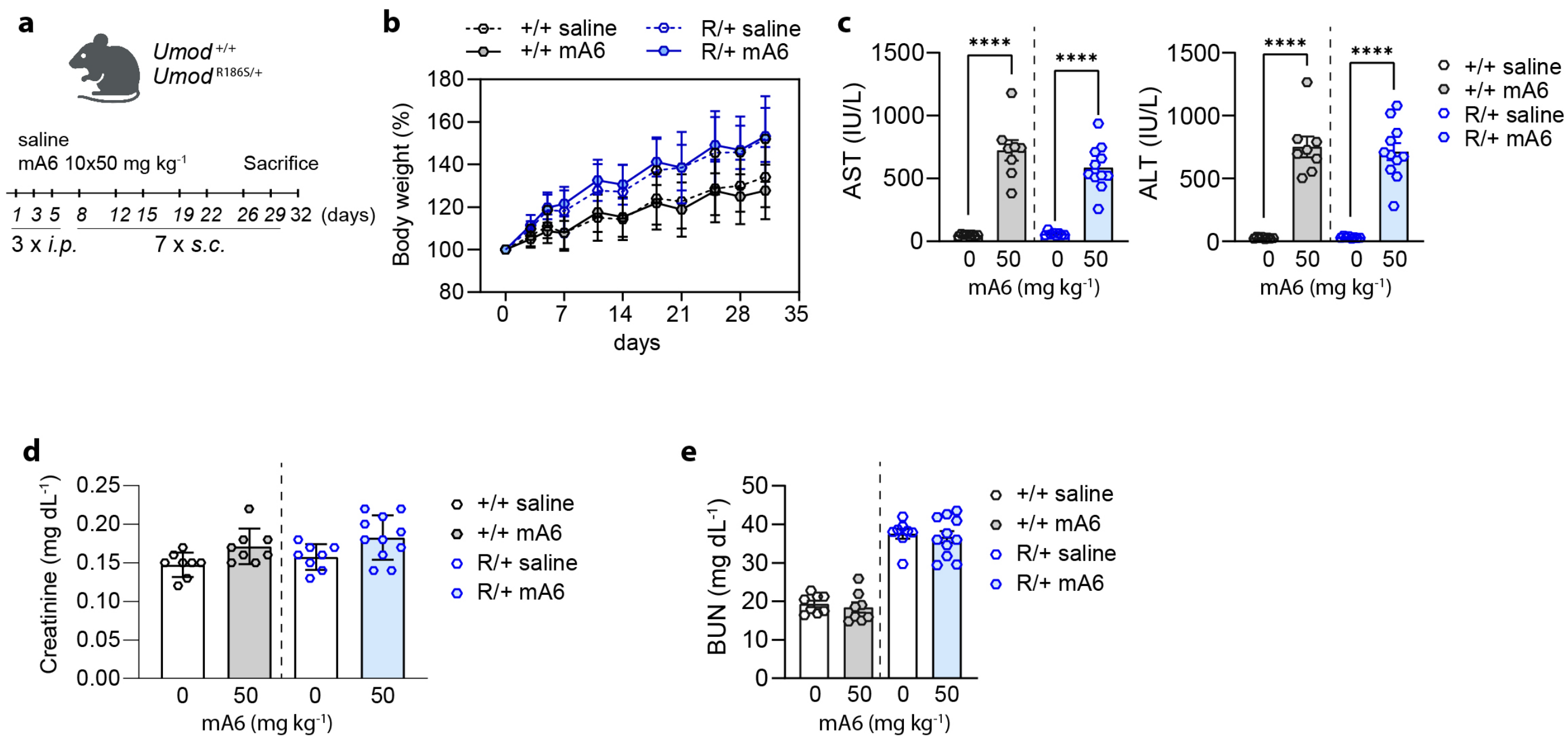

Fig. S9. Safety and tolerability of mA6 in *Umod* mouse models. a) Protocol with three *i.p.* injections 50 mg kg^{-1} and seven *s.c.* injections 50 mg kg^{-1} mA6 or saline, over four weeks in *Umod*$^{+/+}$ and *Umod*$^{R186S/+}$ mice. **b)** Body weight gain throughout 4-week treatment, expressed as % of starting body weight. **c)** Serum levels of AST and ALT measured at the 4-week treatment endpoint. **d)** Serum creatinine levels measured at the 4-week treatment endpoint. **e)** Blood urea nitrogen (BUN) levels in mice at the four-week endpoint blood collection. Data are presented as the mean of biological replicates ± SEM. ****P<0.0001 indicate t test comparison (n = 8-11 animals).

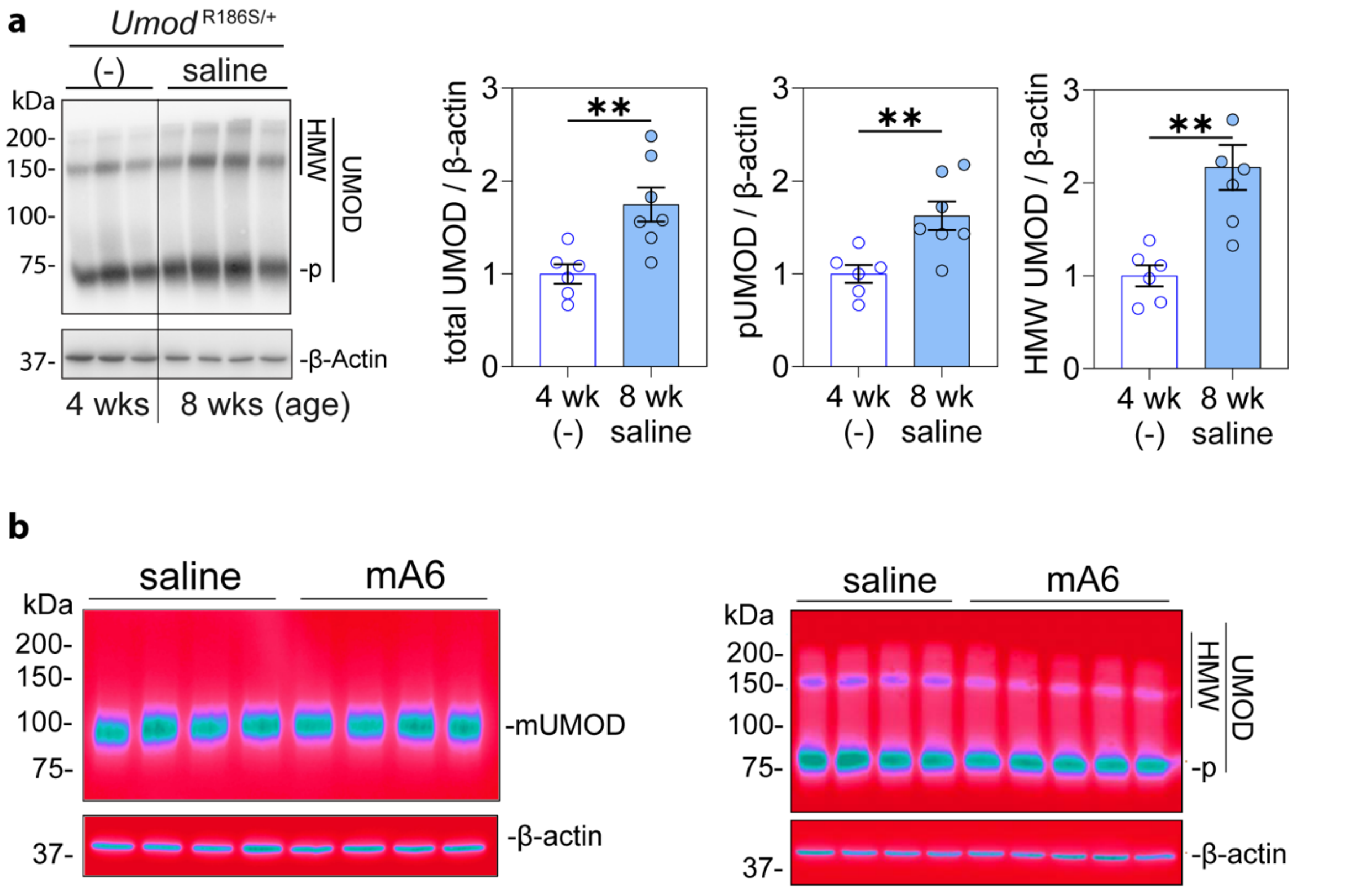

Fig. S10. Progression of UMOD accumulation and effect of mA6-treatment in *Umod*$^{+/+}$ and *Umod*$^{R186S/+}$ mouse kidneys. a) Representative immunoblot and densitometry, comparing the pre-existing uromodulin accumulation in kidneys of *Umod*$^{R186S/+}$ mice 4 weeks old, with that of 4-week saline-treated mice (8 weeks old). **b)** Western blot from (**Fig. 5D**) shown using a false-color LUT (Ice, ImageJ) representation of pixel densities, without modification of the original data.

Table S1. Analytics of purified ASOs by mass spectrometry. Sequences of synthesized ASO candidates targeting human *UMOD* c.553C>A mutation (hA1-20), and targeting the murine *UMOD* c.556C>A mutation (mA6) (**Fig. 1**) (**Fig. 2**). The ASOs were identified by microwalk over the mutation site and their identity of twice HPLC-purified compounds confirmed by liquid chromatography-coupled mass spectroscopy (**Fig. S3**). NTC is a scrambled sequence control ASO used during cell screening (**Fig. 1**) (**Fig. 2**) to rule out sequence-independent effects on uromodulin expression. Masses were calculated using the Oligowizard Nucleic Acid Calculator tool available at http://oligowizard.com/.

ASO ID	5'-3' Antisense Sequence	Observed Mass (g/mol)	Calculated Mass (g/mol)	Error (g/mol)
hA1	TCCAGTACTCGTCCAGGGTG	7181.91	7181.796	0.114
hA2	CTCCAGTACTCGTCCAGGGT	7155.86	7155.809	0.051
hA3	GCTCCAGTACTCGTCCAGGG	7195.00	7194.831	0.169
hA4	TGCTCCAGTACTCGTCCAGG	7170.06	7169.812	0.248
hA5	CTGCTCCAGTACTCGTCCAG	7130.01	7129.782	0.228
hA6	GCTGCTCCAGTACTCGTCCA	7129.94	7129.792	0.148
hA7	TGCTGCTCCAGTACTCGTCC	7120.91	7120.758	0.152
hA8	GTGCTGCTCCAGTACTCGTC	7146.94	7146.75	0.19
hA9	GGTGCTGCTCCAGTACTCGT	7186.85	7186.78	0.07
hA10	CGGTGCTGCTCCAGTACTCG	7171.93	7171.794	0.136
hA11	TCGGTGCTGCTCCAGTACTC	7146.95	7146.765	0.185
hA12	CTCGGTGCTGCTCCAGTACT	7146.93	7146.775	0.155
hA13	ACTCGGTGCTGCTCCAGTAC	7155.93	7155.824	0.106
hA14	TACTCGGTGCTGCTCCAGTA	7170.89	7170.825	0.065
hA15	GTACTCGGTGCTGCTCCAGT	7186.93	7186.81	0.12
hA16	CGTACTCGGTGCTGCTCCAG	7185.85	7185.827	0.023
hA17	CCGTACTCGGTGCTGCTCCA	7159.92	7159.825	0.095
hA18	CCCGTACTCGGTGCTGCTCC	7149.98	7149.793	0.187
hA19	CCCCGTACTCGGTGCTGCTC	7149.94	7149.793	0.147
hA20	TCCCCGTACTCGGTGCTGCT	7164.95	7164.794	0.156
NTC	TCACGGGTTTGGACTGGTAG	7277.11	7276.862	0.248
mA6	GCTGCTCCAGTACTCAGTCA	7140.06	7139.809	0.251
mA6-Cy3	GCTGCTCCAGTACTCAGTCA	7662.24	7664.444	-2.204

Table S2. DNA primers and probes. Primers and probes used in RT-qPCR (**Fig. 1, Fig. 2, Fig. 3** and **Fig. S5**), and SplintR-ligation qPCR (**Fig. 3** and **Fig. 5**), respectively (see materials and methods for details). SplintR ligation qPCR primers were modified to allow for enzymatic ligation between the DNA probes: hA6_B (5'-PO_4^{2-} group) and mA6_A (3'-OH), which ligate after assembly on mA6. SplintR ligation qPCR primers and probes with the prefix "hA6" are compatible with quantification of both hA6 and mA6 due to sequence similarities between these ASOs. "hA6 Cy3" and "hA6 BHQ2" represent a fluorescence – quenching pair allowing for quantification by fluorescence of elongated hA6 Cy3, incorporated into the amplicon.

Application	Name	Sequence 5'-3'	nt	Modification	Complementarity
RT-qPCR	hUMOD F	CGAGTGTCACCTGGCGTAC	19		hUMOD
	hUMOD R	TGTGCTCCAGGAGGGAGATA	20		hUMOD
	mUMOD F	TTGCGAAGAATGCAGGGTAG	20		mUMOD
	mUMOD R	TGGCACTTTCTGAGGGACAT	20		mUMOD
	mUMOD-R186S F	GACCCTGACTGAGTACTGGA	20		mUMOD-R186S
	mUMOD-R186S R	GAGAGCCATTGAGCCACATG	20		mUMOD-R186S
	mUMOD-WT F	GGGGATGGTTGGTACTGT	18		mUMOD-WT
	mUMOD-WT R	ACCATACTCTGTGCTGCG	18		mUMOD-WT
	Lrp2 F	CAGTGGATTGGGTAGCAG	18		Megalin
	Lrp2 R	GCTTGGGGTCAACAACGATA	20		Megalin
	Aqp2 F	TGAGCCTCAAGAAGGGTCTC	20		Aquaporin-2
	Aqp2 R	TCTCCAGAGCTCTCCGTCTC	20		Aquaporin-2
	Casp3 F	GGGCCTGAAATACCAAGTCA	20		Caspase 3
	Casp3 R	TCCCATAAATGACCCCTTCA	20		Caspase 3
	Ppia F	AGGATTCATGTGCCAGGGTG	20		Ppia
	Ppia R	GCCATCCAGCCATTCAGTCT	20		Ppia
	mGAPDH F	TGCACCACCAACTGCTTAGC	20		mGAPDH
	mGAPDH R	GGATGCAGGGATGATGTTCT	20		mGAPDH
	mHspa5 F	CTGAGGCGTATTTGCGAAAG	20		mHspa5
	mHspa5 R	CAGCTGCTGTAGGCTCATTG	20		mHspa5
SplintR ligation qPCR	mA6 A	TTAAACCATGACTGAGTA	18		mA6
	hA6 B	CTGGAGCAGCAACCACGT	18	5'-PO_4^{2-}	mA6/hA6
	hA6 R	ACGTGGTTGCTGCTCCAGT	19		mA6/hA6 lig templ
	hA6 Cy3	CTCCCTCCCTCGATTTAAACCATGGACG	28	5'Cy3	hA6 lig templ compl.
	hA6 BHQ2	AAATCGAGGGAGGGAG	16	3'-BHQ	mA6 Cy3

Table S3. Clinical and biological parameters for *Umod* $^{R186S/+}$ mice after 10-day treatment.

Parameter	*Umod* $^{R/+}$ Baseline (n=15)	*Umod* $^{R/+}$ saline (n=4)	*Umod* $^{R/+}$ mA6 50mg/kg (n=6)	*Umod* $^{R/+}$ mA6 100mg/kg (n=5)
Body weight (BW), g	18.1 ± 1.26	18.8 ± 1.39	18.3 ± 0.88	19.5 ± 1.21
Water intake, $\mu L \cdot min^{-1} \cdot g^{-1}$ BW	0.31 ± 0.01	0.30 ± 0.02	0.30 ± 0.02	0.30 ± 0.02
**Urine**				
Diuresis, $\mu L \cdot min^{-1} \cdot g^{-1}$ BW	0.03 ± 0.01	0.02 ± 0.01	0.02 ± 0.03	0.05 ± 0.02
Na^+, g·g $creat^{-1}$	9.42 ± 0.78	8.86 ± 2.33	8.72 ± 1.34	6.51 ± 0.94
K^+, g·g $creat^{-1}$	38 ± 2.50	28.7 ± 1.34	34.5 ± 2.56	28.6 ± 4.05
Creatinine, mg·dL^{-1}	24.5 ± 1.75	18.4 ± 3.79	23.2 ± 4.15	23.7 ± 3.88
FE_{UA}, %	1.07 ± 0.27	1.17 ± 0.61	0.30 ± 0.07	0.45 ± 0.16
uUMOD mg·g $creat^{-1}$	44 ± 6.88	26.6 ± 7.65	21.02 ± 4.17	35.2 ± 12.9
Osmolality, mOsm·kg^{-1} H_2O	1635 ± 86.1	1051 ± 216	1573 ± 276	1388 ± 66.2
**Plasma**				
Na^+, mmol·L^{-1}	145 ± 0.9	146 ± 2.49	145 ± 0.55	146 ± 1.59
Creatinine, mg·dL^{-1}	0.13 ± 0.023	0.14 ± 0.01	0.12 ± 0.006	0.12 ± 0.004
BUN, mg·dL^{-1}	33 ± 1.84	33.8 ± 3.12	32.4 ± 1.77	31.9 ± 2.38
Uric acid, mg·dL^{-1}	2.96 ± 0.5	6.46 ± 1.28	6.62 ± 0.52	4.75 ± 0.65
Osmolality, mOsm·kg^{-1} H_2O	351 ± 3.8	351 ± 6.51	356 ± 3.12	348 ± 3.78

Values are presented as average ± SEM. n, number of animals; FE_{UA}, fractional excretion of uric acid; BUN, blood urea nitrogen.

Table S4. Clinical and biological parameters for *Umod* $^{R186S/+}$ mice after 4-week treatment.

Parameter	*Umod* $^{R/+}$ Baseline (n=19)	*Umod* $^{R/+}$ saline (n=8)	*Umod* $^{R/+}$ mA6 (n=11)
Body weight (BW), g	14.7 ± 0.43	21.4 ± 1.05	21.3 ± 0.74
Water intake, μL·min^{-1}·g^{-1} BW	0.33 ± 0.01	0.22 ± 0.01	0.21 ± 0.01
**Urine**			
Diuresis, μL·min^{-1}·g^{-1} BW	0.04 ± 0.03	0.03 ± 0.007	0.02 ± 0.004
Na^+, g·g creat^{-1}	5.76 ± 0.18	4.95 ± 0.74	8.48 ± 0.73**
K^+, g·g creat^{-1}	24 ± 0.84	24 ± 2.19	20 ± 1.11
Creatinine, mg·dL^{-1}	43.8 ± 2.37	32.5 ± 2.51	33.9 ± 5
FE_{UA}, %	1.07 ± 0.27	0.45 ± 0.05	0.72 ± 0.08*
uUMOD mg·g creat^{-1}	49.7 ± 4.3	41.6 ± 4.4	53 ± 4.3
Osmolality, mOsm·kg^{-1} H_2O	1906 ± 80	1381 ± 67	1519 ± 183
**Plasma**			
Na^+, mmol·L^{-1}	145 ± 0.9	144 ± 1.14	144 ± 0.50
Creatinine, mg·dL^{-1}	0.13 ± 0.023	0.16 ± 0.006	0.18 ± 0.009*
BUN, mg·dL^{-1}	33 ± 1.84	37.5 ± 1.25	36.7 ± 1.53
Uric acid, mg·dL^{-1}	2.96 ± 0.5	5.64 ± 0.53	5.47 ± 0.53
Osmolality, mOsm·kg^{-1} H_2O	351 ± 3.8	341 ± 1.6	339 ± 2.4

Values are presented as average ± SEM. *$P < 0.05$, **$P < 0.01$ versus age-matched saline. n: number of animals, FE_{UA}: fractional excretion of uric acid, BUN: blood urea nitrogen.

Table S5. Clinical and biological parameters for *Umod*$^{+/+}$ mice after 4-week treatment.

Parameter	*Umod*$^{+/+}$ Baseline (n=15)	*Umod*$^{+/+}$ saline (n=8)	*Umod*$^{+/+}$ mA6 (n=7)
Body weight (BW), g	16.67 ± 0.30	21.5 ± 0.87	21.1 ± 1
Water intake, µL·min^{-1}·g^{-1} BW	0.28 ± 0.008	0.22 ± 0.01	0.21 ± 0.02
**Urine**			
Diuresis, µL·min^{-1}·g^{-1} BW	0.04 ± 0.004	0.02 ± 0.004	0.01 ± 0.001
Na^+, g·g creat^{-1}	5.75 ± 0.27	4.40 ± 0.31	7.23 ± 0.29****
K^+, g·g creat^{-1}	24.3 ± 0.81	23 ± 1.08	19 ± 0.82**
Creatinine, mg·dL^{-1}	39 ± 2.28	46.6 ± 2.97	45.5 ± 2.33
FE_{UA}, %	0.55 ± 0.12	1.10 ± 0.22	0.83 ± 0.15
uUMOD mg·g creat^{-1}	36.4 ± 5.24	76.1 ± 10.02	75.9 ± 7.47
Osmolality, mOsm·kg^{-1} H_2O	1768 ± 88	1940 ± 93	1968 ± 69
**Plasma**			
Na^+, mmol·L^{-1}	146 ± 1.3	147 ± 1.3	145 ± 0.43
Creatinine, mg·dL^{-1}	0.17 ± 0.03	0.15 ± 0.01	0.17 ± 0.01
BUN, mg·dL^{-1}	21.5 ± 1.68	19.3 ± 0.85	18.4 ± 1.4
Uric acid, mg·dL^{-1}	2.36 ± 0.40	4.48 ± 0.56	6.81 ± 1.12
Osmolality, mOsm·kg^{-1} H_2O	341 ± 5.6	332 ± 1.7	329 ± 3.9

Values are presented as average ± SEM. **$P< 0.01$, ****$P< 0.0001$ versus age-matched saline. n: number of animals, FE_{UA}: fractional excretion of uric acid, BUN: blood urea nitrogen
